# Wearable Devices in Scoliosis Treatment: A Scoping Review of Innovations and Challenges

**DOI:** 10.3390/bioengineering12070696

**Published:** 2025-06-25

**Authors:** Samira Fazeli Veisari, Shahrbanoo Bidari, Kourosh Barati, Rasha Atlasi, Amin Komeili

**Affiliations:** 1 Department of Biomedical Engineering, University of Calgary, Calgary, AB T2N 1N4, Canada; samira.fazeliveisari@ucalgary.ca; 2 Department of Orthotics and Prosthetics, School of Paramedical and Rehabilitation Sciences, Mashhad University of Medical Science, Mashhad 9919191778, Iran; bidarish@mums.ac.ir (S.B.); baratik@mums.ac.ir (K.B.); 3 Endocrinology and Metabolism Research Center, Endocrinology and Metabolism Clinical Sciences Institute, Tehran University of Medical Sciences, Tehran 1411713119, Iran; rashaatlasi@gmail.com

**Keywords:** scoliosis, wearable devices, artificial intelligence, smart braces, rehabilitation, telemedicine, treatment

## Abstract

Scoliosis is one of the most common spinal deformities, which affects millions of people worldwide. Bracing and physiotherapy exercises represent the first-line, non-invasive approaches for managing scoliosis. In recent years, the use of wearable devices has spread as a novel approach to the treatment of scoliosis. However, their effectiveness in treatment planning and outcomes has not been thoroughly evaluated. This manuscript provides a scoping review of the classification and application of wearable devices and the role of artificial intelligence (AI) in interpreting the data collected by wearable devices and guiding the treatment. A systematic search was carried out on Scopus, Web of Science, PubMed, and EMBASE for studies published between January 2020 and February 2025. A total of 269 studies were screened, and 88 articles were reviewed in depth. Inclusion criteria encompassed articles focusing on wearable devices integrated into smart braces, rehabilitation systems for scoliosis management, AI and machine-learning (ML) applications in scoliosis treatment, virtual reality (VR), and telemedicine for scoliosis care. The literature shows that the use of wearable devices can enhance scoliosis treatment by improving the efficiency of braces and enabling remote monitoring in rehabilitation programs. However, more research is needed to evaluate user compliance, long-term effectiveness, and the need for personalized interventions. Future advancements in artificial intelligence, microsensor technology, and data analytics may enhance the efficacy of these devices, which can lead to more personalized and accessible scoliosis treatment.

## 1. Introduction

The human spine provides structural support for movement and protects the spinal cord. Conditions such as adolescent idiopathic scoliosis (AIS), a three-dimensional spinal deformity, may lead to functional limitations and significantly impact health and quality of life [1]. AIS usually emerges during growth spurts, affecting approximately 2–4% of children under 16 years worldwide, with progression often occurring during periods of rapid growth [2].

Non-surgical treatments, including bracing, exercise therapy, chiropractic care, electrical stimulation, and observation, remain the first-line approach for managing scoliosis, particularly for mild and moderate scoliosis curves. In recent years, the use of wearable devices in the diagnosis and treatment of scoliosis has been expanding [3]. Wearable devices, equipped with sensors, are designed to monitor human activity and health in real time, tracking various physiological and biochemical parameters. In scoliosis management, these systems allow for continuous spine motion monitoring and posture assessment, supporting remote rehabilitation, biofeedback, and individualized treatment strategies [3]. Among non-surgical treatment interventions, bracing is the most commonly prescribed modality, particularly for skeletally immature individuals with coronal plane curvature (measured by the Cobb method) of more than 10 degrees [4], aimed at halting curve progression during periods of growth. A range of scoliosis braces have been developed over the years, including rigid thoracolumbosacral orthoses (TLSO) [5,6], dynamic braces that allow some movement while applying corrective forces [7], and nighttime-only braces designed to be worn during sleep [8]. These braces are typically custom-fitted and prescribed with specific usage protocols by clinicians.

The advantages of bracing and other non-surgical interventions are substantial. When prescribed and used appropriately, they can delay the need for surgery until skeletal maturity or, in many cases, avoid surgical intervention altogether. Bracing has also been associated with psychosocial benefits, such as reduced anxiety around future surgery, and physiological benefits, including improved postural control and spinal alignment during growth [9,10]. Additionally, dynamic or night-time bracing options offer patients increased flexibility and comfort, potentially improving adherence.

Despite these benefits, non-surgical treatment faces several challenges. Treatment success is highly dependent on user adherence, yet compliance often declines over time, especially among adolescents. Moreover, frequent follow-up visits are required for monitoring progress, adjusting brace fit, and reinforcing patient engagement, posing logistical and financial burdens for families. The variability in brace types, wear-time protocols, and clinical expertise has also contributed to inconsistent outcomes in terms of curve stabilization and patient satisfaction. These challenges underscore the need for standardized treatment protocols, improved wearable monitoring tools, and patient-centered innovations to enhance the effectiveness and accessibility of non-surgical scoliosis care.

In recent years, advancements in wearable sensors, additive manufacturing, and AI have paved the way for a new generation of scoliosis braces with novel features, such as computer-aided design and manufacturing (CAD/CAM), pressure sensors for wear monitoring, remote monitoring, and AI-driven customization. Moreover, applications of digital platforms, such as telemedicine and VR, in the treatment of scoliosis have been catalyzed since the COVID-19 pandemic, with benefits including shorter wait times, easier access to healthcare professionals, and high patient satisfaction levels [11]. These innovations adapt to the patient’s daily movement patterns and monitor real-time adherence, aiming to enhance treatment efficacy and improve patient compliance.

Despite these developments, there remains a need to synthesize evidence on the characteristics of modern bracing interventions and their mechanisms of action. For instance, recently, AI-based data analytics have gained popularity in brace design and brace treatment, particularly in the diagnosis of curve progression and classification, interpretation of sensor data, and control systems of robotic exoskeletons. However, their effectiveness in supporting clinicians with their treatment decisions, generalization potential in correcting the scoliosis curve, and patient compliance remain under investigation. Also, there is a notable gap in the literature regarding the challenges in employing digital and remote therapeutic platforms in scoliosis treatment.

This review aims to map and summarize recent advancements in scoliosis bracing technology, focusing on smart scoliosis braces, integration of digital tools in brace design and manufacturing, AI-driven methods in scoliosis care, and digital and remote platforms. By evaluating current evidence and identifying gaps in intervention reporting and outcome assessment, this review seeks to inform both clinical practice and future research directions in non-surgical scoliosis management.

## 2. Materials and Methods

### 2.1. Study Design

A scoping review methodology was chosen for this review to keep the data extraction broad and to be able to provide a summary of the existing literature concerning the objectives and identify knowledge gaps where further research may be needed. Broad Exploration of the Topic and Rapidly Evolving Technology would make a formal systematic review or meta-analysis misleading and lead to choosing a scoping review for this topic.

In this scoping review, we have used a combination of MeSH terms and free text words pooled through Boolean operators (‘AND’ and ‘OR’) in the PubMed, Scopus, Web of Science, and EMBASE databases. A systematic search was carried out on 29 March 2025 to find all papers in the field of wearable devices in scoliosis treatment. The detailed search strings for each database are provided in the Supplementary Material file. This scoping review adhered to the guidelines for PRISMA-ScR (Preferred Reporting Items for Systematic Reviews and Meta-Analyses Extension for Scoping Reviews).

### 2.2. Search Strategy

In the present scoping review, the selection of articles was based on precise inclusion and exclusion criteria. Narrative synthesis was used to categorize the included studies into four major themes: (1) sensor-integrated orthotics, (2) electromyography (EMG)-based systems, (3) digital and remote therapeutic platforms, and (4) AI/ML-integrated rehabilitation technologies.

The inclusion criteria were limited to the studies published between January 2020 and March 2025. All articles not relevant to the wearable devices in scoliosis treatment, not available in English, and with the full text, and other types of reports (theses, books, and conference papers) were excluded. We used EndNote (Version 21) for reference management and automatic removal of duplicates.

Given the exploratory and descriptive nature of a scoping review, which emphasizes mapping the breadth of available evidence rather than judging study quality, formal risk-of-bias assessment was not performed. To limit potential bias during study selection and data extraction, two reviewers (S.F.V./A.K.) independently reviewed the records as part of the article selection process, and disagreements were resolved through discussion or a third reviewer (Sh.B.). Only peer-reviewed, full-length articles were retained, minimizing misinterpretation stemming from incomplete reports. These measures helped ensure a transparent and reliable evidence map despite the absence of a formal quality assessment. After removing papers published before January 2020, duplicates, theses, books, conference papers, reports, and non-English papers, the remaining papers were initially screened for titles and abstracts, grading each study as eligible/not eligible/might be eligible at each stage. At the next step, the full text versions of the remaining articles were further analyzed. In cases where any disagreement occurred, a third reviewer was involved.

### 2.3. Study Selection

**Figure 1:**
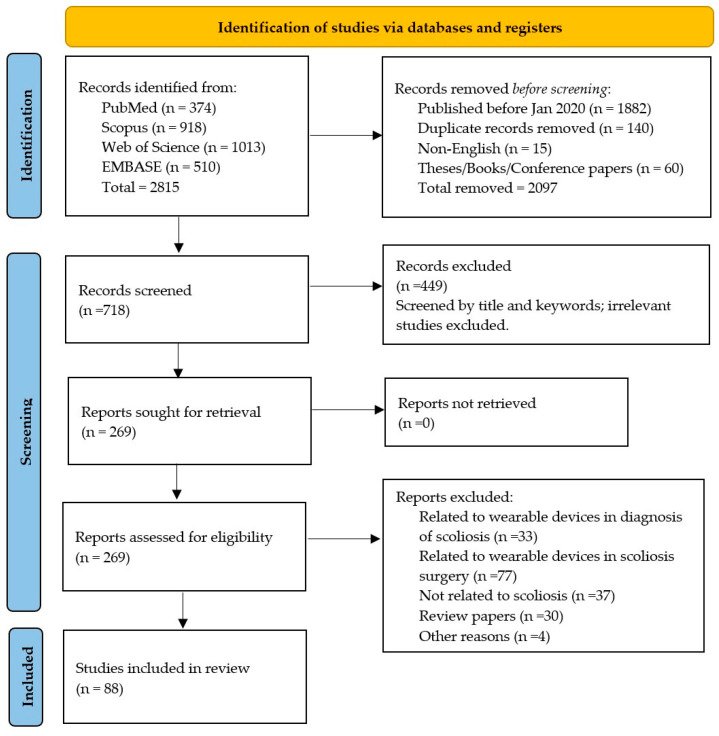
PRISMA-ScR flowchart for article identification and screening.

Of the 2815 identified studies in the PubMed (*n* = 374), Scopus (*n* = 918), Web of Science (*n* = 1013), and EMBASE (*n* = 510) databases, 1882 articles published before January 2020 were excluded. Next, 140 duplicates, 15 non-English language papers, and 60 theses, books, and conference papers were removed. The remaining 718 articles were screened by their title and keywords, and 449 articles were removed. The full text of the remaining 269 articles was analyzed by the reviewers, and 88 papers were considered for this scoping review. The screening process of the selected publications is described in the PRISMA-ScR flowchart (Figure 1). The list of excluded studies after full-text screening, along with justifications, is provided in Table S2 in the Supplementary Material.

## 3. Results

A total of 88 articles from the last five years (2020–2025) were included in this review. The role of Wearable Devices in brace Treatment of scoliosis was identified in four main areas: Sensor-Integrated Wearable and Orthotic Technologies, EMG-based wearables, Digital and Remote Therapeutic Platforms, and Integration of AI and ML for Personalized Rehabilitation (Table 1, Table 2, Table 3 and Table 4). The primary outcomes of interest included objective indicators of scoliosis assessment and treatment efficacy, such as Cobb angle reduction, wear-time compliance, biomechanical feedback, user engagement, and system accuracy. All reported outcomes relevant to the review objectives were extracted from each study, regardless of time point or measurement method. If multiple outcomes were reported, those directly aligned with the core technology categories (e.g., EMG signals for wearables, AI prediction accuracy for telemedicine tools) were prioritized. No statistical data conversions or handling of missing data were required. All data were charted as reported in the original studies.

**Figure 2:**
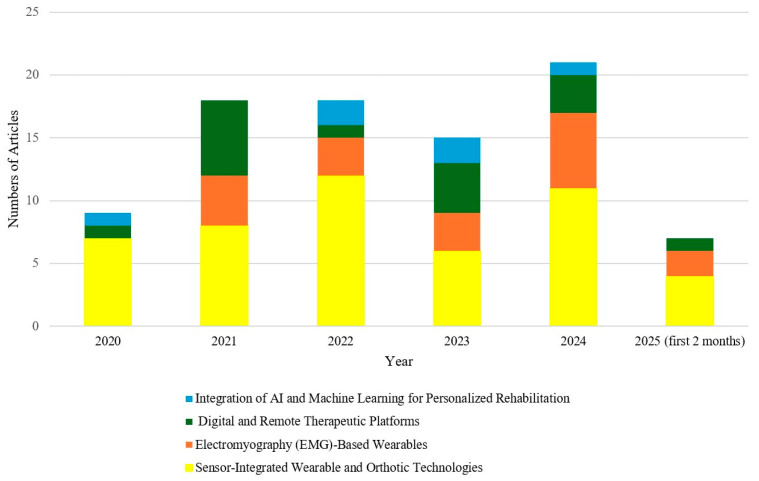
The main themes of reviewed articles across the published years.

**Figure 3:**
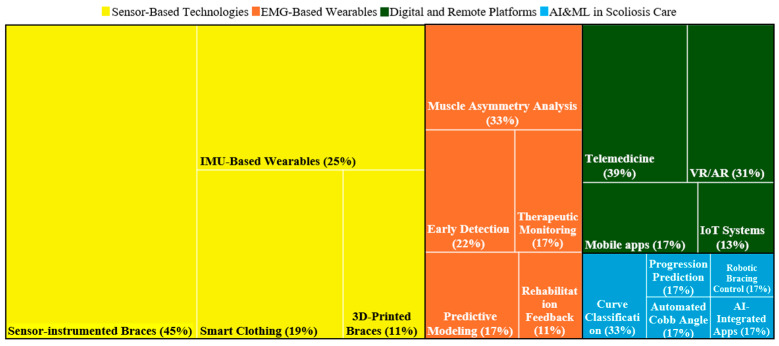
The percentage distribution of reviewed publications in four identified categories.

**Figure 4:**
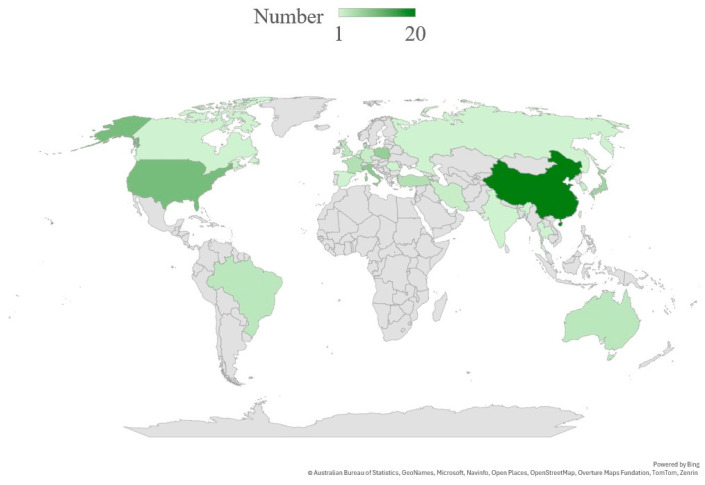
A heat map of the world map representing the number of first-authored publications, considered in the present review, from each country.

Figure 2 shows the yearly distribution of published articles from 2020 to 2024 and the first two months of 2025 across four categories, showing a peak in 2024 with the highest contributions from Sensor-Integrated Wearable and Orthotic Technologies. Extrapolating the January–February 2025 publication rate linearly to the full year in Figure 2 will suggest a pronounced uptick, with sensor-integrated wearable and orthotic technologies poised to more than double their 2024 output and remain the fastest-growing research theme in this domain. Within each of the four overarching themes, we further partitioned the literature into discrete research streams to highlight the most active subtopics (Figure 3). In the Sensor-Based Technologies theme, sensor-instrumented braces account for the largest share of publications (45%), followed by inertial measurement unit (IMU)-based wearables (25%), smart clothing (19%), and 3D-printed braces (11%). The EMG-Based Wearables theme is led by studies on muscle asymmetry analysis (33%) and early detection (22%), with additional interest in therapeutic monitoring (17%), predictive modelling (17%), and rehabilitation feedback (11%). For Digital and Remote Platforms, research is heavily concentrated on telemedicine (39%) and VR/AR applications (31%), with secondary attention to mobile apps (17%) and IoT-enabled systems (13%). Finally, within AI and ML for Scoliosis Care, curve classification dominates (33%), while progression prediction, automated Cobb-angle measurement, robotic bracing control, and AI-integrated mobile applications each constitute 17% of the studies. Figure 4 illustrates the geographic distribution of published articles between 2020 and 2025 related to wearable devices in scoliosis treatment, highlighting China and the United States as the leading contributors in this field, followed by European countries, including Italy.

### 3.1. Sensor-Integrated Wearable and Orthotic Technologies

This review section highlights recent innovations in bracing and garment-based interventions for scoliosis. Of the 88 papers reviewed, 48 articles addressed the topic of sensor-integrated wearable and orthotic technologies. These were categorized into four groups: 3D-printed bracing, smart brace systems, smart clothing embedded with sensors, and IMU-based wearables. The characteristics of the included studies are presented in Table 1.

Those studies focused on 3D-printed braces (*n* = 5) developed and evaluated customized scoliosis braces using additive manufacturing. The effectiveness of the proposed braces was assessed through clinical trials, simulation modeling, and observational research, with sample sizes ranging from single-case studies to large cohorts. Most prototypes used materials such as polyethylene terephthalate glycol-modified (PETG) and flexible polymers, aiming to optimize stiffness, comfort, and mechanical performance. Pilot studies [12,13,14] demonstrated that 3D-printed braces could provide comparable biomechanical outcomes to traditional orthoses, with added benefits such as reduced manufacturing time, lower costs, and improved patient comfort. Clinical evaluations reported improvements in Cobb angle, postural stability, and user satisfaction [14,15]. Innovative design was highlighted in some studies, including braces with asymmetric stiffness [15]. These innovative designs are aimed at providing dynamic, non-invasive alignment correction. Although one of these studies lacked clinical validation, its engineering evaluations demonstrated promising biomechanical feasibility. Martin et al. confirmed the feasibility of a 3D-printed sensor for real-time pressure monitoring, though its clinical effectiveness requires further in-field evaluation [16].

Studies that introduced smart braces (*n* = 22) demonstrated various applications made possible by smart orthotic technologies. For instance, some studies used smart braces for real-time monitoring of corrective pressure [5,17], wear-time adherence [4,18,19], posture, and activity [10,20]. Other studies used smart braces to enhance their performance through force adjustment mechanisms [21,22,23,24], applied soft materials to enhance comfort and compliance [25,26], and used finite element (FE) modeling in CAD/CAM workflows [6,8,27]. Various sensor technologies were integrated into braces, including textile-based [4,18,26], dielectric [16], temperature [28], and combined force and temperature sensors [19]. The various types of sensors and their applications in wearable devices for scoliosis treatment are categorized and briefly described below:

- Pressure sensors:
  These sensors are commonly embedded in smart braces to monitor the magnitude and distribution of corrective forces applied to the torso. They are used to provide real-time feedback for clinicians and patients and improve treatment adherence and adjustment accuracy [5,17,18].
- IMUs:
  IMUs consist of accelerometers and gyroscopes and are used to assess posture, gait, and spinal curvature during daily activities. They are particularly useful in wearable belts, braces, and clothing for tracking range of motion and balance [29,30,31,32].
- Surface Electromyography (sEMG) Sensors:
  sEMG sensors are employed to measure paraspinal muscle activity and asymmetry. They are helpful in identifying neuromuscular dysfunctions, predicting scoliosis progression, and evaluating the effects of physical therapy [33,34,35,36].
- Temperature sensors:
  These are primarily used to detect brace wear-time compliance. Temperature fluctuations help distinguish between active use and non-use of the brace during daily routines or sleep [19,28,37].
- Textile-based or flexible sensors:
  Capacitive or piezoresistive sensors integrated into smart garments allow for continuous pressure and postural monitoring. Their soft, adaptable design enhances comfort and long-term usability [26,38,39].
- Capacitive and dielectric sensors:
  These printed or stretchable sensors monitor localized force distribution in braces. They provide high-resolution mapping of internal pressures and are promising tools for custom brace adjustments [16,19].

The application of smart clothing systems in scoliosis braces is advancing significantly by offering real-time posture monitoring, improved biomechanical feedback, and enhanced user comfort. Of the nine reviewed studies, three demonstrated the effectiveness of wearable garments embedded with sensors in detecting and correcting postural deviations [3,38,40]. In addition to monitoring, smart clothing facilitated biomechanical improvements and adaptivity in treatment. Taping-based interventions showed potential in correcting trunk kinematics [41]. Adaptive garment models designed using 3D skeleton referencing and instrumented with pressure-sensing intelligent wearables demonstrated accurate force monitoring, fast response times, and strong correlations with radiograph measurements, enhancing clinicians’ ability to personalize treatment [39,42,43,44]. Caviedes et al. [3] used a custom-designed triangular array of stretchable sensors attached to torso landmarks for the recognition of four therapeutic exercise executions. Using a single-class classifier algorithm, they achieved a sensitivity and specificity of 88.5% and 100%, respectively, enabling real-time compliance monitoring of patients under no supervision.

Studies focused on IMU-based wearable systems (*n* = 12) explored their utility in scoliosis diagnosis, treatment monitoring, postural evaluation, and gait analysis. IMUs were integrated into braces, belts, or sensor arrays to measure spinal curvature, brace wear time, postural balance, and gait patterns under real-life conditions. Several studies studied the effectiveness of IMUs for real-time feedback and activity classification, with more than 99% accuracy [29,30]. Clinical applications showed beneficial results such as improved brace compliance (from 7% to 90%), identification of pressure drop trends [29], and the effects of scoliosis-specific exercises on postural balance improvements [9]. Other studies used IMU data to evaluate spinal motion restriction across different braces [31], model 3D spinal curvature [32], and assess motor control or gait regularity in AIS patients [45,46]. Kim et al. used ML in combination with sensor fusion to enhance spinal curvature classification without requiring personalized calibration [30]. Other studies utilized flexible sensor tapes [47] and temperature sensor evaluations for optimal brace wear tracking [37]. These devices provide real-time data to track scoliosis progression and guide rehabilitation, while integration with ML enhances predictive accuracy for curve progression. By complementing traditional methods, IMU wearables advance personalized, data-driven care in scoliosis management.

**Table 1:** The characteristics of the included studies (*n* = 48) in the field of sensor-integrated wearable and orthotic technologies.

Citation	Objective	Methodology	Key Findings and Conclusions
3D-printed braces for individualized treatment			
Redaelli, Alexander Storm (2020) [12]	Feasibility and cost-effectiveness of 3D-printed vs. thermoformed scoliosis braces.	Comparative study (N = 1)	FDM 3D printing with PETG offers a cost-effective, customizable alternative to thermoforming for scoliosis braces, with superior stiffness, comfort, and short-term durability.
Storm, Redaelli et al. (2022) [13]	Stability comparison of FDM, conventional, and unbraced conditions using sensors.	Pilot study evaluating the production process Clinical comparison (N = 10)	3D-printed PETG scoliosis braces proved cost-effective and customizable, offering comparable stability to conventional braces and eliminating the need for plaster casting.
Costantini, Redaelli et al. (2025) [14]	To assess whether 3D-printed braces offer advantages or disadvantages over conventional braces.	Clinical pilot study (N = 10)	3D-printed braces, providing customizable support through additive manufacturing without negatively affecting gait dynamics, showed no significant differences in mobility compared to conventional braces, with both similarly limiting trunk motion and reducing AP acceleration while preserving other gait metrics.
Li, Yang et al. (2022) [15]	3D-printed patient-specific brace with asymmetric stiffness for targeted corrective force.	Design and simulation, clinical application (N = 1)	The brace featured asymmetric stiffness (concave: 145.88 N/mm, convex: 35.96 N/mm) with higher corrective force on the convex side (33.53 N vs. 3.27 N). Cobb angle reduced from 49° to 42.4° after 6 months.
Martin, Gugel et al. (2024) [16]	To develop a flexible, printed dielectric sensor grid for real-time pressure monitoring inside scoliosis braces.	Material and sensor development Proof-of-concept testing (N = 0)	A 34 wt.% graphite–silicone mix achieved optimal conductivity (0.32 kΩ) with stable resistance under 50% strain. Square-electrode capacitance grids enhanced sensitivity 10×. Pressure monitoring in scoliosis braces improves but still requires further calibration and testing due to uneven and lateral pressure issues.
Smart bracing systems			
Fregna, Raccagni et al. (2024) [1]	Influence of personal and clinical factors on brace adherence in AIS patients.	Cross-sectional study (N = 638)	Brace adherence in AIS was highest among younger, less skeletally mature females, with optimal compliance at 19–22 h/day, supporting the role of psychosocial factors and sensor-based monitoring.
Erzurumluoglu, Ozlem et al. (2024) [4]	To examine the pressure applied to the brace by integrating a textile-based pressure sensor array.	Laboratory-based feasibility study	The textile-based capacitive sensor system showed high accuracy, low error, and stable pressure mapping, proving suitable for real-time monitoring in scoliosis braces.
Tymins, Zaborowska-Sapeta et al. (2022) [5]	Presents a prototype of the intelligent brace	Prototype development and clinical testing (N = 1)	The smart brace prototype, using graphene-based sensors, enabled real-time tracking of corrective forces (~150 N) and patient compliance, effectively detecting irregular usage and supporting mobile-based monitoring.
Guy, Labelle et al. (2021) [6]	To assess the effectiveness of CAD/CAM brace design, with and without simulations.	RCT CAD/CAM: N = 61, CAD-FEM: N = 59	CAD-FEM and CAD-only braces showed similar clinical outcomes, with finite element method (FEM) improving design efficiency but offering no significant advantage in correction, adherence, or quality of life.
Yahyaiee Bavil and Rouhi (2020) [8]	To evaluate night-time Providence brace’s effectiveness in reducing the Cobb angle.	Combined experimental and computational study. (N = 1)	The Providence brace significantly reduced Cobb angle via FE modeling and clinical results, with strong predictive accuracy (4.4% error), highlighting its effectiveness in preventing scoliosis progression.
Fuss, Ahmad et al. (2021) [17]	To develop a cost-effective but accurate pressure sensor system for TLSOs.	Development, material testing, and experimental testing (N = 2)	The developed low-cost sensor system demonstrated viability for long-term pressure monitoring in scoliosis braces, showing minor hysteresis from foam liners while maintaining ~12% RMSE accuracy, with peak pressures (up to 0.135 MPa) during deep inspiration, primarily in thoracolumbar regions.
Gesbert, Colobert et al. (2021) [18]	Reliability analysis of a textile sensor system for the measurement of brace pressure.	Prospective pilot study, Calibration tests, and clinical validation (N = 4)	The (Smart T-Shirt, STS) prototype showed high accuracy (r^2^ = 0.99) and reproducibility (r^2^ > 0.9), effectively detecting posture-based pressure changes, proving reliable for real-time brace pressure monitoring and design optimization.
Zou, Zhou et al. (2025) [19]	To evaluate the feasibility of an integrated force-temperature for monitoring AIS brace compliance.	Prospective pilot study (N = 12)	Patients overestimated brace wear time, but a dual-sensor system (force + temperature) achieved 92.3% accuracy in detecting true wear, enabling early detection of non-compliance for timely interventions.
Catalina, Robert, and Calin (2020) [10]	To design a smart orthosis that can detect postural changes in the variation of spine curvature.	Design and evaluation research (N = 0)	The study presents a smart orthosis integrating inertial and flex sensors that enhances posture awareness and rehab outcomes via real-time mobile feedback.
Gaume, Pietton et al. (2020) [20]	To assess the mean walking distance per day in AIS patients using smartphone-based pedometers.	Prospective observational study. Control group: N = 25, AIS: N = 19	Braced AIS patients walked significantly more than untreated peers and controls, with no link between Cobb angle and activity, showing AIS does not limit activity; smartphone pedometers offer a low-cost monitoring solution.
Lin, Lou et al. (2020) [21]	To compare the effectiveness of the automated pressure-adjustable orthosis (PO) and conventional orthosis (CO) for the treatment of AIS.	RCT PO group: N = 11 CO group: N = 12	The pressure-optimized orthosis outperformed conventional braces with greater correction, no curve progression at 1-year follow-up, and significantly improved compliance and wear time, enhancing scoliosis management without affecting quality of life.
Ray, Nouaille et al. (2023) [22]	Proposes a robotic solution that provides greater mobility and adapts the procedure to the patient.	Validating tests and feasibility (N = 1)	A 2.5 kg modular robotic brace using Stewart-Gough platforms showed precise trajectory tracking and adaptive motion, offering improved comfort and motion accuracy over traditional rigid braces for scoliosis correction.
Lim, Mak et al. (2024) [23]	To assess the efficacy of a 3D customized over-corrective brace, “ScoliBrace,” an orthosis treatment for AIS	Prospective pilot study (N = 30)	ScoliBrace wear showed dose-dependent efficacy, reducing Cobb angle by 0.794° per extra hour worn, with optimal adherence at 15–18 h/day, effectively halting progression and avoiding surgery in most cases.
Marchese, Du Plessis et al. (2024) [24]	To evaluate short- and medium-term trunk extensor and abdominal endurance in AIS patients using ScoliBrace^®^ and ScoliBalance^®^ treatment.	Retrospective study (N = 33)	The combined Scoli-Brace^®^ and ScoliBalance^®^ treatment significantly enhanced trunk and abdominal endurance in AIS patients over time, with many achieving the 180 s maximum hold duration by follow-up. The approach particularly strengthened trunk extensors without causing muscle weakening.
Ali, Fontanari et al. (2022) [25]	Presents finite element analysis (FEA) of an active soft brace	Computational modeling and simulation (N = 0)	The validated FE model showed that the active soft brace reduces Cobb angle and pressure, providing a comfortable, corrective alternative to rigid braces.
Cakmak, Cegindir et al. (2022) [26]	A posture-supporting garment for scoliosis, developed using pattern engineering and textile structures.	Develop and test a prototype (N = 1)	Prototype A, a soft exosuit brace, matched rigid orthoses in Cobb angle correction (up to 10.7%) while providing superior comfort, increased wear time, and high satisfaction through optimized design and materials.
Farhadiyadkuri and Zhang (2023) [27]	A Multi-Body-FE Simscape model and an analytical model of the AIS bracing treatment are created.	Computational Modeling and Simulation (N = 0)	This study introduced the first MB-FE Simscape model with NPIC control for AIS bracing, achieving <6.5% stiffness error and superior tracking precision (<1% stiffness/damping error, 0.88–3.28% pose error) over traditional methods.
Zapata, Virostek et al. (2024) [28]	To measure night-time brace adherence using temperature sensors and its impact on curve progression.	Prospective case series (N = 122)	Night-time use of the Providence brace effectively prevented AIS progression, especially in patients with single or smaller curves and high adherence, with only 6% requiring surgery, lower than rates seen in full-time bracing.
Redchen, Vissarionov et al. (2021) [48]	Evaluated yearly outcomes of trunk orthoses post-surgery in children with congenital spinal deformities.	Prospective clinical study (N = 25)	Functional-corrective orthoses provided up to 60% curve correction post-surgery, with thoracic improvements partly sustained and lumbar curves regressing, supporting extended lumbar orthosis use until skeletal maturity.
Ali, Fontanari et al. (2022) [49]	Presents an active soft brace that maintains spinal mobility while applying corrective elastic forces.	Modeling and validation experiments (N = 0)	The TSA-powered brace delivered safe, effective corrective forces (0–6 kPa) with accurate control (RMSE: 1.74 mm), preserving mobility and enabling prolonged, low-power scoliosis treatment comparable to rigid braces.
West et al. (2022) [50]	To assess the accuracy of the Scolioscreen (smartphone with an application) compared to the Scoliometer.	Diagnostic Accuracy Study (N = 50)	Scolioscreen showed high accuracy and inter-rater reliability (ICC ≥ 0.93) for ATR measurement, offering a low-cost, user-friendly tool for reliable at-home scoliosis screening, even by untrained parents.
Zapata, McIntosh et al. (2023) [51]	To evaluate the effects of adding physiotherapeutic scoliosis-specific exercises (PSSE) to night-time bracing.	Prospective comparative study (N = 115)	PSSE combined with night-time bracing outperformed bracing alone in managing thoracolumbar/lumbar curves < 35°, reducing curve progression, improving Cobb angle stability, and lowering full-time bracing need in AIS patients.
Smart clothing embedded with sensors			
Caviedes, Jammula et al. (2020) [3]	Wearable sensor system for home therapy providing real-time feedback to improve exercise accuracy.	Design and simulation, clinical application. Sensor array with mobile app integration	The wearable sensor system, using custom garments and harnesses, demonstrated 70–100% sensitivity and perfect specificity in detecting exercises, enabling real-time feedback for posture correction and therapy adherence.
Anwary, Cetinkaya et al. (2021) [38]	To develop the Smart-Cover for real-time sitting posture and asymmetry monitoring using pressure sensors.	Laboratory-based feasibility study (N = 10)	Smart-Cover detected sitting asymmetry with ±4% accuracy over a 4.2–8.4 kg pressure range, provided real-time mobile-app feedback, offering a cost-effective solution to promote ergonomics and prevent posture-related disorders.
Simegnaw, Teyeme et al. (2022) [40]	To develop a smart shirt with motion sensors for real-time monitoring of trunk posture and bending angles during cycling.	Validating tests and feasibility (N = 1)	The smart shirt accurately measured trunk bending angles (8–35°), with 99% of postures within ergonomic limits. Its CA-74 threads remained reliable after 17 washes, enabling precise, real-time posture monitoring to support cycling performance, injury prevention, and broader ergonomic applications.
Yağcı, Turgut, and Yakut (2020) [41]	Scapular taping effects on 3D shoulder and thoracic motion in AIS daily activities.	Prospective intervention study with pre-post testing (N = 24)	Elastic scapular taping improved scapular orientation (external and upward rotation), as well as humeral and trunk kinematics during functional tasks, supporting dynamic stability and upper limb function in adolescents with scoliosis.
Lee, Yip et al. (2023) [39]	To develop a smart textile with silicone-embedded fiber Bragg grating (FBG) sensors for real-time bracing pressure monitoring in AIS patients.	A PIMA cotton undergarment with embedded FBG sensors was developed and validated.	Silicone-embedded FBG sensors provided reliable and sensitive force measurements (up to 10 N, R^2^ > 0.90, ICC = 0.97), improving brace pressure monitoring in AIS through better stability, skin compatibility, and enhanced fit and comfort for improved clinical outcomes.
Lee, Wang et al. (2025) [42]	To develop body pressure mapping knitwear (BPMK) with FBG sensors for force monitoring.	Sensor-embedded textile testing, Force measurement + FEA simulation	The FBG-equipped BPMK system provided accurate real-time force monitoring with strong agreement to FEA and X-ray data, enhancing comfort, adaptability, and MRI compatibility, safety, compliance, and scoliosis treatment outcomes.
Mosleh, Abtew et al. (2021) [43]	To create adaptive clothing for women with scoliosis that adjusts to spinal deformity progression.	Validating tests and feasibility (N = 1)	The 3D geometrical model enables automated, adaptive garment design for AIS patients using skeleton-based referencing and ease allowances, enhancing fit and comfort while minimizing scanning and manual adjustments.
Liu, Zhang et al. (2025) [44]	To investigate soft brace effects on balance, proprioception, and textile design in AIS.	Pilot study, experimental trial, (N = 10), 3D scanning, EOS, motion capture	The PCG soft brace improved postural alignment and proprioception—especially in thoracic/lumbar regions—highlighting its potential for managing mild scoliosis, though personalization and long-term evaluation remain essential.
Xuan, Lei et al. (2024) [52]	To develop a smart wearable system for real-time brace monitoring and comfort assessment.	Experimental Sensor-integrated 3D-printed orthosis	The intelligent system offers reliable, real-time monitoring of orthopedic pressure and comfort with <2 s latency and <2% error, enhancing adherence, personalization, and clinical decision-making in scoliosis bracing.
Inertial Measurement Unit (IMU)-based wearables			
Dehzangi, Bache et al. (2021) [29]	To develop a smart scoliosis brace system for unsupervised monitoring of wear time, tightness, and activity.	Prospective, single-center, observational study, Boston-type TLSO with wearable sensors	The system enables accurate, remote, context-aware monitoring for scoliosis bracing, achieving >99% activity classification accuracy, improving compliance from 7% to 90%, and detecting a 33% force drop, supporting early identification of poor fit and timely personalized re-fitting.
Kim, Hwang et al. (2024) [30]	To develop a wearable system (WLSCMS) for real-time monitoring of spine curvature using IMU sensors combined with ML.	Design and simulation (N = 20), Smart waistband, ML used to classify spinal curvature	The WLSCMS system offers a user-friendly, accurate wearable for lumbar monitoring, achieving >99% classification accuracy without individual calibration and outperforming single-sensor setups, with strong clinical potential pending further validation.
Selthafner, Liu et al. (2021) [9]	To evaluate the effectiveness of PSSE on postural balance in AIS patients by measuring the center of pressure (COP) metrics.	Prospective, intervention study, N = 6 (AIS patients), 12-week PSSE program, pre- and post-imaging DIERS 4D	After PSSE, measurable postural improvements were observed through COP and forefoot pressure changes—15% increase in anterior-posterior COP, 25% decrease in medial-lateral COP, and improved symmetry, supporting dynamic pressure analysis as a valuable tool for evaluating AIS therapy outcomes.
Fercho, Krakowiak et al. (2022) [31]	To evaluate three spinal braces’ effectiveness in restricting spinal motion using IMUs during daily tasks.	Comparative study (N = 10, healthy adults), IMU-based motion analysis	Spinal orthoses restrict motion task-specifically; TLSO excels in sitting/walking, Hohmann in lifting, with IMUs revealing significant performance differences.
Mak, Liang et al. (2023) [32]	To develop and evaluate a neural network using IMUs for 3D spinal curvature estimation.	Design and simulation, clinical application (N = 15, healthy participants)	The IMU-based neural network accurately estimated spinal curvature (0.26 cm error) and Cobb angles, offering a cost-effective, real-time alternative to X-rays for scoliosis monitoring, with potential for clinical, home, and biofeedback use.
Farella, Vanzini et al. (2022) [45]	Investigation of motor control alterations in children with idiopathic scoliosis using clinical assessments and IMU-based metrics.	Motor and gait analysis in 34 scoliosis children using ABC-2 and IMUs vs. healthy controls.	Children with idiopathic scoliosis show impaired motor control, slower and more variable gait, and reduced stability; IMU-based non-linear metrics enable early detection and support intervention planning.
Gan, Liu et al. (2024) [46]	Evaluating gait symmetry, regularity, and head balance control in AIS patients using wearable accelerometers.	Comparative study (N = 34; 17 scoliosis patients, 17 healthy controls)	AIS patients exhibit reduced head and lumbar vertical motion and lower head regularity, indicating impaired walking balance; wearable accelerometers offer effective, non-invasive tools for real-world gait assessment and monitoring.
Hong, Wang et al. (2022) [47]	To develop a flexible, non-invasive sensor tape with integrated accelerometers for real-time spinal curvature assessment.	Design and validation study (N = 15) Flexible sensor tape with 30 MEMS accelerometers	The novel sensor tape demonstrated 99% accuracy and strong correlation with Vicon in spinal angle measurements, offering a reliable, low-cost, and reusable tool for accurate, non-radiative spinal monitoring with strong clinical and ergonomic potential.
Wardell, Jayasuriya et al. (2022) [37]	To determine the best sensor placement and temperature threshold for accurately measuring scoliosis brace wear time.	Comparative study Seven Orthotimer and five iButton sensors are embedded in the spinal brace.	Optimal brace wear detection was achieved using abdomen and axilla sensors, with iButton preferred clinically and highest accuracy from uninsulated placement on the anterior abdominal wall using a 26 °C threshold.
Żurawski, Śliwiński et al. (2023) [53]	To present a 3D photogrammetric model for monitoring spinal curvature changes in children with postural disorders.	Clinical pilot study (N = 211, spinal deformity patients; 101 controls)	Photogrammetric systems enable sensitive, non-invasive monitoring of pediatric postural rehabilitation, revealing significant improvements in trunk imbalance, pelvic tilt, and spinal curvature, while highlighting the need for individualized, dynamic therapy assessment.
Asano, Inami et al. (2024) [54]	To study dynamic spinal and lower limb alignment during gait in adult spinal deformity (ASD) patients compared to static posture using IMUs.	Comparative study (N = 34, ASD patients), IMUs used to track gait-phase spinal and joint changes	IMU data show that in ASD patients, forward trunk tilt during gait results from lumbar flexion and pelvic anteversion, with worsened posture compared to standing, emphasizing the importance of dynamic assessment.
Truong, Matsumoto et al. (2024) [55]	To establish guidelines for safely managing pediatric scoliosis with implanted programmable devices (IPD).	Modified Delphi consensus study (N = 25 experts)	Experts reached over 94% consensus on safe use of neuromonitoring and MCGRs in IPD patients, supporting device deactivation during surgery and confirming that, with precautions, standard tools are safe for scoliosis care.

### 3.2. EMG-Based Wearables

sEMG technology has emerged as a critical tool for early detection and progression monitoring. The 19 reviewed papers in the field of EMG-based wearables focused on studying the role of wearable devices that monitor physiological and neuromuscular signals in scoliosis treatment. Table 2 presents the characteristics of the included studies in this field. 

Paraspinal muscle asymmetry, characterized by elevated EMG activity on the convex side of spinal curves, is a hallmark of scoliosis [33,34]. This asymmetry correlates strongly with lumbar Cobb angles, particularly at the L_3_ vertebral level, suggesting localized biomechanical stress [33]. Larger curves (>20° Cobb angle) exhibit destabilizing paraspinal muscle activity, whereas smaller curves demonstrate compensatory stabilization, suggesting a threshold beyond which muscular contributions transition from adaptive to pathological [35]. In pediatric populations (ages 7–8), sEMG amplitude and frequency profiles correlate with curvature location, direction, and severity, enabling pre-radiographic identification of at-risk individuals [36]. Functional deficits in scoliosis patients, including reduced gait velocity, shorter stride length, and elevated gait deviation indices, were consistently observed compared to healthy controls [56]. These impairments are directly attributable to spinal deformity rather than curve type or etiology, emphasizing the systemic neuromuscular consequences of structural misalignment [57].

Exercises and corrective devices that target localized regions influence muscle activation patterns, supporting their inclusion in rehabilitation protocols. For instance, TheraBand-assisted regimens [58] and self-corrective postures [59] resulted in superior paraspinal muscle activation compared to traditional unilateral approaches.

These innovations, combined with machine learning-driven predictive analytics, bridge diagnostic and therapeutic gaps, offering scalable solutions for precision care. For instance, support vector machines (SVM) enhanced diagnostic precision by integrating lumbar EMG ratios and anthropometric data, achieving 85% classification accuracy [60]. The synergy of sEMG with 3D spinal imaging further refined monitoring, enabling dynamic, non-invasive surveillance [34].

**Table 2:** The characteristics of the included studies (*n* = 18) in the field of Electromyography (EMG)-Based Wearables.

Citation	Objective	Methodology	Key Findings and Conclusions
Garg, Gupta et al. (2021) [33]	To compare gait patterns among AIS, congenital scoliosis, and healthy controls to identify differences in gait parameters.	Prospective Cohort Study with EMG and radiographic analysis. 79 AIS Patients	Lumbar and thoracolumbar scoliosis exhibit convex-side EMG asymmetry and coronal imbalance, with asymmetry strongly correlated with the lumbar Cobb angle—most notably at L3—whereas thoracic scoliosis shows no significant muscle activity asymmetry.
Zhang, Bao et al. (2024) [34]	To evaluate the clinical effectiveness of sEMG combined with a spine 3D data system.	Original research Participants: 10 AIS patients (6 thoracic, 4 lumbar curves)	sEMG combined with 3D motion data enables accurate, non-invasive assessment of muscle asymmetry and Cobb angle, especially in lumbar scoliosis, with strong correlation to EOS imaging (r = 0.971), supporting dynamic scoliosis monitoring.
Wong, Shayestehpour et al. (2024) [35]	To investigate spinal muscle roles in AIS—stabilization vs. progression—using TES, radiography, and EMG.	Cross-sectional study, 45 AIS (subgroups: TES = 5, EMG = 6).	In small-curve AIS, spinal muscles contribute to stability, while in larger curves (>20°), thoracic erector spinae (TES) activation may worsen deformity. Higher summed EMG ratios indicate corrective muscle function, whereas lower ratios are associated with progression risk.
Wilczynski and Karolak. (2021) [36]	To explore the relationship between the sEMG frequency of erector spinae and scoliosis curve characteristics in children.	Observational Participants: 244 children, 103 with scoliosis, 141 with scoliotic posture.	sEMG patterns in AIS reveal gender-specific differences and abnormal erector spinae activity, suggesting CNS dysfunction, with potential for standardized sEMG profiles in early detection and prevention
Park, Ko et al. (2021) [56]	Study muscle imbalance (EMG), leg length difference, and scoliosis curve types in AIS patients.	Prospective Comparative Study, groups: AIS (*n* = 20), congenital scoliosis (*n* = 20), healthy controls (*n* = 20).	AIS and congenital scoliosis patients exhibit similar gait abnormalities driven by spinal deformity, with convex-side muscle dominance highlighting the need for concave-side strengthening and leg length assessment.
Wang, Jiang et al. (2022) [57]	To investigate neuromuscular synergy patterns in the back muscles of AIS patients using high-density EMG.	Prospective comparative study with 10 AIS patients and 10 healthy controls	AIS patients demonstrate asymmetric muscle activation during dynamic tasks, particularly with Cobb angles >20°, including increased convex-side activity and reduced dominant-side control. High-density EMG and synergy analysis effectively reveal these neuromuscular adaptations, supporting their use in guiding targeted therapies.
Haksever, Soylu et al. 2024) [58]	To analyze spinae muscle activation during three types of 3D scoliosis-specific elongation exercises.	Prospective cohort study, 24 adolescents with AIS	TheraBand resistance elicits strong paraspinal activation in AIS patients, with LES showing greater side differences, supporting its use in 3D-targeted exercises for bilateral muscle rebalancing.
Vongsirinavarat, Kao-ngampanich et al. (2023) [59]	Comparing the paraspinal muscle activity during three self-corrective positions and the habitual standing in AIS.	Cross-sectional observational study 33 adolescents with AIS	Corrective positions enhance EMG ratios compared to habitual standing, with the O1 position most effective for thoracic curves and symmetrical correction optimal for lumbar curves. These self-corrective exercises improve muscle activation across all PUMC curve subtypes.
Liang, Yip et al. (2022) [60]	To analyze the relationship between physical features and EMG signals in scoliosis patients using ML.	Cross-sectional study with 106 participants (73 scoliosis patients, 33 controls)	Lumbar EMG asymmetry and paraspinal imbalance in AIS patients support sEMG biofeedback and ML-based early detection, with SVM achieving 85% prediction accuracy using ATR, height, and EMG ratio as key features.
Wilczynski and Karolak. (2021) [61]	To analyze the relationship between scoliosis features and sEMG amplitude of the erector spinae in young school children.	Cross-sectional study, N = 251 children, 41%: scoliosis, 56%: scoliotic posture, 3%: normal posture	Thoracic curvatures and sex-related curve direction differences were most common, with elevated erector spinae sEMG amplitudes correlating with curve characteristics, supporting sEMG as a non-invasive tool for early scoliosis detection and monitoring.
Banno, Yamoto et al. (2022) [62]	To compare muscle activity in ASD patients and age-matched controls using surface EMG.	Prospective case series study. ASD Group: 14 Control Group: 8	Improved post-op muscle activity in ASD patients suggests pre-op sEMG abnormalities may reflect CNS dysfunction contributing to scoliosis onset, supporting standardized sEMG profiles for early detection and prevention.
Asada, Miura et al. (2023) [63]	Examined time-based changes in spinal alignment and muscle activity during walking in ASD patients.	Retrospective study 26 ASD patients	In ASD patients, walking worsens spinal alignment with increased SpSVA and compensatory cervical PVM and Gmax activity; degeneration limits thoracic/lumbar PVM response, and quadriceps fatigue may reduce walking endurance.
Fan, To et al. (2023) [64]	To analyze EMG differences in paravertebral muscles and compare EMG patterns between AIS patients and healthy individuals.	Prospective cohort study with 6-month follow-up. 534 girls (267 AIS, 267 age-matched controls).	AIS patients showed significant EMG asymmetry, with rms-EMG ratios strongly predicting curve progression (AUC: 96% thoracic, 90% lumbar), making asymmetry at AV during back extension a powerful early indicator of scoliosis progression.
Fan, Wang et al. (2024) [65]	To develop a pressure and sEMG sensor array into a scoliosis brace to enhance treatment efficiency.	Proof-of-concept study integrating wearable sensors with ML.	The MXene-based sensor array enabled real-time scoliosis monitoring with high durability, accurate sEMG and pressure sensing, and 100% motion classification, supporting brace optimization and curve progression prediction.
Ng, Duncombe et al. (2024) [66]	Compared activation in resisted vs. unresisted trunk extension in adolescents with and without scoliosis.	Cross-sectional study Participants: 44 females (AIS = 24, controls = 20).	No group-level difference in peak activation between unresisted and resisted tasks, but high individual variability suggests both are needed to accurately assess paraspinal MVC in adolescents.
Wang, Xia et al. (2024) [67]	Analyzed activation to assess neuromuscular characteristics of scapular stabilizers in AIS patients.	Cross-sectional study, observational study. Participants: 17 AIS, 19 controls	AIS patients exhibited impaired scapular neuromuscular control with reduced concave-side AMAs and altered muscle timing, suggesting a need for targeted rehab to enhance stability and motor control.
Muraoka, Hasegawa et al. (2025) [68]	To determine whether spinal correction surgery in ASD patients reduces muscle activity and fatigue.	Prospective cohort study, Participants: 16 ASD patients, 16 healthy controls	Preoperative ASD patients had elevated muscle activity that decreased post-surgery, reflecting reduced fatigue and improved alignment, HRQOL, and pain, with I-EMG confirming enhanced postoperative muscle efficiency.
Wang, Wang et al. (2025) [69]	Paraspinal muscle activity comparison in AIS vs. healthy individuals for rehab optimization.	Case-control study Participants: 12 AIS patients. 10 healthy controls (age-matched).	AIS patients showed greater muscle asymmetry and reduced sitting activity; targeted Schroth exercises improved paraspinal activation, while erect sitting may worsen S-curve asymmetry.

### 3.3. Digital and Remote Therapeutic Platforms

Digital and remote therapeutic platforms have emerged as effective virtual and remote solutions for enhancing scoliosis therapy and patient engagement [70]. Sixteen studies explored the development and application of telemedicine, mobile apps, and digital tools, such as VR, for scoliosis monitoring, screening, and therapy enhancement (Table 3).

Nine studies assessed telemedicine applications in scoliosis care. For instance, Culpepper et al. [11] used a web-based survey for follow-ups in early-onset scoliosis. Several mobile apps automatically assess posture, range of motion (ROM), or trunk asymmetry using standard cameras or 2D image processing [71]. Clinical pilot studies examined apps for monitoring brace adherence and wellbeing, showing the potential to improve patient safety and personalize care [72]. BLE- and IoT-based systems enabled real-time, wireless control and data upload to cloud platforms for remote scoliosis management [73]. Other studies assessed scoliosis-specific apps across usability and functionality criteria. For instance, Bottino et al. proposed guidelines for app selection [74]. Seven studies assessed the use of VR, augmented reality (AR), and video game-based systems in enhancing therapy outcomes and patient engagement. VR-assisted physical therapy showed significant improvements in spinal curvature, postural control, and respiratory function across various patient groups, including adolescents, children, and adults with degenerative scoliosis [75]. Overall, these immersive technologies enhanced therapeutic outcomes, better adherence, and individualized care strategies in both clinical and home settings.

**Table 3:** The characteristics of the included studies (*n* = 16) in the field of digital and remote therapeutic platforms.

Citation	Objective	Methodology	Key Findings
Günther, Schober et al. (2023) [70]	Explored motivational barriers and tools to improve home-based scoliosis therapy.	Cross-sectional survey study (Patients: N = 72, Therapists: N = 30)	Most scoliosis patients under-exercised, but motivation—especially in older adults—improved with peer support, assistive devices, and digital tools, highlighting the value of tech-integrated strategies for effective home-based therapy.
Culpepper, Murphy et al. (2024) [11]	To assess how telehealth use evolved in early-onset scoliosis (EOS) care during and after the COVID-19 pandemic.	Web-based survey (N = 191, Pediatric Spine Study Group members)	Telehealth use for early-onset scoliosis surged during the pandemic, with bracing and curve monitoring most compatible; common virtual exam methods included back inspection and gait observation, while legal and reimbursement issues continue to limit broader adoption. Telehealth is a lasting, valuable complement to in-person EOS care, especially for follow-ups.
Moreira, Teles et al. (2020) [71]	To the use of smart mobile apps with ML for measuring human posture and ROM.	Design and simulation Mobile app prototype using TensorFlow and PoseNet	AI-powered smartphone apps accurately measured posture and ROM by identifying key landmarks, offering a low-cost, accessible tool for musculoskeletal monitoring in clinical and home settings.
Martínez-Borba, Suso-Ribera et al. (2021) [72]	To test the feasibility of a mobile app to monitor brace adherence and wellbeing in AIS.	Clinical pilot study (N = 40 adolescents) 90-day trial using a mobile app	Since this is a study protocol, outcome data are not available. The anticipated outcomes include high adherence and usability rates, effective detection of brace-related issues, and increased treatment safety through early interventions based on app-generated alerts.
Xia, Hou et al. (2021) [73]	To develop a spinal orthopedic system using BLE and IoT for remote control and monitoring of spinal deformity treatment.	Design and simulation, BLE-enabled growing rod system integrated with mobile app and cloud server	The mobile app-enabled system accurately monitored spinal elongation and adherence with real-time cloud connectivity, offering a personalized, low-power solution to enhance AIS care and reduce clinical workload.
Bottino, Settino et al. (2023) [74]	To evaluate digital tools for scoliosis management and propose a framework to assess their features and usability.	Comparative analysis study (N = 7 tools)	Most scoliosis apps measure ATR via motion sensors; only one estimates Cobb angle. Functions vary, and a selection guideline was proposed based on OS, features, and user skill. Digital scoliosis tools enhance monitoring and engagement. The evaluation framework supports clinicians and patients in selecting the right app, encouraging smarter scoliosis care.
Akazawa, Torii et al. (2021) [76]	To create an accessible, low-cost mobile app with a standard 2D camera for screening scoliosis.	Design and simulation, clinical application, Trunk asymmetry detection,	A 2D camera-based mobile app reliably detects trunk asymmetry for scoliosis screening using image analysis, offering a scalable solution for resource-limited settings.
Yates, Rehman et al. (2021) [77]	Demonstration of wearable-based Human digital twin (HDT) for remote post-op scoliosis recovery monitoring.	Case study (N = 1)	Recovery times varied across physiological metrics (e.g., 3 weeks for sleep, 19 weeks for resting HR). By 5 months post-op, the patient exceeded baseline step count by over 800 steps/day, indicating improved mobility and activity.
Yuan, Shi et al. (2025) [78]	To compare digital scoliosis-specific exercise therapy using the Healbone system with traditional in-person care for AIS.	RCT, (N = 128 patients), Digital care (HIRS-supported home therapy) vs. usual care	The digital care group showed significantly greater Cobb angle improvement and pelvic tilt correction, along with higher engagement and daily exercise adherence compared to usual care. Home-based PSSE via the HIRS system offers an effective and accessible alternative for managing AIS.
Taslimipour, Rojhani-Shirazi et al. (2021) [75]	Evaluation of a VR dance training program’s effect on thoracic kyphosis angle and respiratory function (FVC, FEV1).	Randomized controlled trial (32 women). Compare traditional therapy vs. therapy + Xbox Kinect-based VR dance training.	Both groups showed improvements in kyphosis angle and FVC. The VRRT group demonstrated greater improvements than the RT group in all outcomes: Kyphosis angle: −13.23° vs. −7.33°, FEV1 increase: +8.06% vs. +1.81%, FVC increase: +7.93% vs. +3.31%. VRRT had larger effect sizes and statistical power. VR enhances engagement, motor control, and proprioception.
Kandasamy, Bettany-Saltikov et al. (2021) [79]	Evaluation of a VBAR app’s impact on physiotherapy students’ learning of spinal anatomy and deformities.	A crossover study (N = 74 students) comparing traditional learning to VBAR	VBAR significantly improved students’ understanding of spinal anatomy, increased engagement, and clarified misconceptions compared to traditional methods, with high usability and strong support for collaborative and self-directed learning.
Moraes, Cardoso et al. (2022) [80]	To evaluate whether immersive virtual reality (IVR) enhances the effectiveness of postural therapy for scoliosis treatment.	Randomized controlled trial (N = 22 children) Comparison of VR-assisted therapy vs. traditional therapy	The VR training group showed significantly greater improvements in postural stability, tolerance time, and proprioceptive control than the control group, with enhanced engagement contributing to better consistency and endurance. Immersive VR showed promise as a physiotherapy adjunct, though sustained motivation and long-term effects require further study.
Misterska, Górski et al. (2023) [81]	To develop a VR system using biometric avatars to assess body image perception in adolescent girls with AIS	Design and simulation, comparative study, Use of “Avatar Scoliosis 3D” VR app with 3D body scans	AIS patients often overestimated their deformity, with avatar-based VR revealing individual body image distortions correlated with Cobb angle, offering objective insight into body perception issues and even post-surgery. The VR-based tool provided a promising method for both clinical assessment and psychological support.
Nam, Park et al. (2023) [82]	To evaluate the Smart-Bar Device (SBD) with AR for improving posture accuracy in scoliosis exercises.	Clinical pilot study (N = 10 healthy adults + AIS patients)	The SBD demonstrated high validity (r = 0.836–0.988) and significantly improved posture accuracy in scoliosis patients using AR guidance, with real-time visual and auditory feedback enhancing movement precision. AR-based feedback improves the accuracy of stretching exercises and may benefit home-based rehabilitation for scoliosis patients.
Moraes, Palmeira et al. (2024) [83]	To assess the effectiveness of VR-supported postural therapy versus traditional methods using baropodometric analysis.	RCT, (N = 22) Comparison of VR-assisted therapy vs. traditional therapy	While both groups improved posturally, the VR-assisted group showed greater gains in balance and foot pressure metrics, with significant reductions in asymmetries, attributed to enhanced visual feedback and interactive engagement in the VR environment.
Wan, Mak et al. (2024) [84]	To evaluate the effectiveness of a motion-sensing video game in improving posture and alignment in adults with adult degenerative scoliosis (ADS).	Clinical pilot study (N = 10 older women with ADS) 6-week training program using a posture-controlled video game	Participants improved sagittal alignment (notably head and C7 position). While spine length slightly decreased, shoulder and pelvis symmetry improved over the 6 weeks. Muscle activity remained imbalanced, indicating habitual movement patterns. Although some effects diminished over time, video games promoted engagement and could support long-term posture correction when used frequently or in conjunction with home-based systems.

### 3.4. Integration of AI and Machine Learning for Personalized Rehabilitation

In the last section of the present work, we reviewed six studies focusing on integrating AI and ML for scoliosis care. In these studies, advanced computational techniques were integrated into machine control systems and data analysis for personalized care (Table 4). AI methods were used for curve classifications, assessment, and monitoring. For instance, markerless 3D surface scanning and neural networks achieved high accuracy in scoliosis severity classification [85]. One study reported the use of dynamic segmental calibration (DSC) and AI-integrated mobile apps for spinal mobility and Cobb angle assessment [7]. Petrosyan et al. [86] analyzed motion capture and ultrasound data using advanced data analytics for continuous scoliosis therapy monitoring. AI methods were also used in controlling a patient-specific robotic exoskeleton and offered precise, adaptive corrective forces [87]. These studies support the potential of using sensor-driven, AI-enhanced tools for personalized, effective scoliosis therapy and follow-up.

Beyond integration with wearables, AI methods are revolutionizing the treatment of scoliosis by analyzing biomedical images, particularly spine X-ray images, for classification and curve measurements. Multiple studies have demonstrated the potential of deep learning (DL) models to assess spinal curvature from X-rays and predict curve progression with accuracy levels surpassing those of experienced clinicians. While most DL models required the examiner to manually identify and mark some landmark features from the X-ray, some newer models achieved a fully automated implementation. Li et al. [88] trained a neural network framework based on the transformer mechanism with a ResNet encoder to predict the Cobb angle from spinal X-rays. The model was trained on 18,000 X-rays and achieved an accuracy of above 92% in identifying Cobb angle with an error of less than 5°, surpassing manual methods in consistency and speed. 

Aside from developing new ML models, existing open-source AI models, such as ChatGPT, Microsoft Bing, and Scholar AI, demonstrated unmatched rater concordance and excellent performance in classifying curve type from descriptive data. Despite some struggles in classifying moderate curves, the study showed enormous potential of AI in analyzing descriptive medical data for scoliosis classification. 

**Table 4:** The characteristics of the included studies (*n* = 6) in the field of Integration of AI and ML for personalized rehabilitation.

Citation	Objective	Methodology	Key Findings and Conclusions
Yahara, Tamura et al. (2022) [2]	To develop and evaluate a deep convolutional neural network (DCNN) model for predicting curve progression in AIS patients.	Pilot study (N = 58)	The DCNN model outperformed surgeons in predicting scoliosis progression (69% accuracy, AUC 0.70), with improved performance via transfer learning and focus on spinal regions per Grad-CAM, enabling early, objective, and personalized treatment planning for mild, immature cases.
Kaczmarek, Pulik et al. (2023) [7]	Evaluation of lumbar torsional mobility via real-time DSC for physiotherapy planning.	Clinical pilot study, (N = 55 healthy adults)	Mean rotation to the right: 4.78° ± 2.24°; to the left: 2.99° ± 1.44° (*p* < 0.0001). Max rotation: right 11.35° ± 3.33°, left 7.42° ± 1.97°. No significant sex-based differences. The DSC device offers real-time spinal mobility assessment, supports low back pain therapy planning, and shows reliable performance for future clinical use.
Rothstock, Weiss et al. (2020) [85]	To develop a semi-automatic ML method using 3D surface topography for scoliosis severity and treatment group classification.	Design and simulation, clinical application (N = 50 AIS patients), neural network trained	The model achieved 90% accuracy (80% sensitivity, 100% specificity) in classifying scoliosis severity and up to 75% accuracy in simplified ALS group classification, with reduced performance when distinguishing similar curve patterns.
Petrosyan, Makaryan et al. (2024) [86]	To propose and validate a targeted safe model using MoCap and ultrasound for continuous IS therapy monitoring.	Clinical pilot study (N = 6 AIS patients) 6-month PSSE and Boston brace rehabilitation program	Reductions in Cobb angle (X-ray: from 36.2° to 23.6°, *p* < 0.001; MoCap: from 34.6° to 21.4°, *p* = 0.0014). SRS-22 score improved from 2.6 to 3.9 (*p* = 0.002). Strong negative correlation between muscle thickness/mass and Cobb angle (SMM: R = –0.96). L3–L4 muscle gains were linked to curve reduction, supporting personalized, segment-targeted PSSE and bracing.
Farhadiyadkuri, Popal et al. (2022) [87]	To develop and validate a patient-specific robotic spine exoskeleton with adaptive control to overcome limitations of traditional AIS bracing.	Designed a robotic exoskeleton using 3 Stewart-Gough platforms with 18 actuators, employing inverse dynamics and adaptive impedance control.	MRAIC provided superior position tracking and smoother, safer interaction than conventional control, enabling the exoskeleton to deliver precise corrective forces at T4, T7, and T11 while maintaining performance under uncertainty and sensor noise. The robotic exoskeleton with MRAIC enables safer, adaptive, closed-loop scoliosis treatment.
Li, Qian et al. (2024) [88]	To assess an AI app for Cobb angle measurement, validating its accuracy against manual picture archiving and communication system (PACS) methods.	Design and simulation, clinical application (N = 601 scoliosis patients) Mobile app for Cobb angle estimation.	The app demonstrated excellent consistency with PACS (ICC > 0.97), low mean absolute error (~2°), and high accuracy across scoliosis severities, outperforming PACS in repeatability for Cobb angle measurements. The AI app offered a fully automated, portable solution for clinical and remote scoliosis follow-up.

## 4. Discussion

### 4.1. Key Findings

The convergence of advanced manufacturing techniques, wearable technology, and digital health platforms is setting a transformative trajectory for scoliosis treatment in the context of patient-centered, data-informed spinal care. Recent advancements in scoliosis treatment have centered around improving the accuracy of diagnosis, personalizing care, and enhancing the effectiveness of conservative treatments such as bracing. Geographically, China, the United States, and some European countries were the leading contributors to the field (Figure 4), indicating a concentration of research capacity and technological innovation in the treatment of scoliosis.

The reviewed studies reveal a growing trend toward advanced manufacturing methods with new materials that enable the development of braces that are adaptable to the patient’s body and behavior, while being more efficient to produce and comfortable to wear. Three-dimensionally-printed braces demonstrated the feasibility of delivering individualized, low-cost orthoses with mechanical performance comparable to traditional thermoformed devices [15,16]. PETG and flexible polymers are common materials due to their balance of stiffness, durability, and comfort [12,13,14].

Smart bracing systems with sensor integration enabled continuous monitoring of corrective forces, pressure distribution, and wear-time adherence. Optimization through computational modeling and the use of soft materials significantly improved clinical outcomes, patient comfort, and compliance [6,8,25,26,27]. Innovative brace designs incorporating asymmetric stiffness and pressure-adjustable pads led to better Cobb angle control and higher treatment adherence [6,15,21]. Smart bracing also provided more accurate monitoring and addressed overestimation of wear-time in self-reported data [19], highlighting the importance of digital tracking for accurate adherence assessment. Moreover, the application of muscle electrical stimulation and EMG helped identify neuromuscular imbalances that may influence the development and progression of AIS [3] Moreover, muscle electrical stimulation and EMG helped identify neuromuscular imbalances that may influence the development and progression of AIS. These tools provide insights into muscle activity patterns, asymmetries, and fatigue that may contribute to spinal deformity.

The significant potential of integrating smart brace technologies and AI was highlighted in the new braces to enhance conservative care. While effective in halting curve progression, traditional rigid bracing systems often suffer from limitations, including poor comfort, adherence challenges, and lack of real-time adaptability. The integration of AI into smart braces is transforming this landscape by empowering the treatment with data-driven decision making, accurate and early diagnosis, patient-specific brace design, and real-time progression monitoring. Smart braces of the future will not only correct spinal deformities but also serve as intelligent therapeutic ecosystems. AI-powered braces will be capable of learning patient-specific parameters, monitoring spinal alignment continuously, and responding adaptively to postural changes. The use of wearable sensors embedded in braces to capture kinematic and physiological data generates big data that requires advanced analyses to exploit information not guaranteed to be obtained from classical data analyses. Moreover, radiographic data does not include various indicators of scoliosis progression, including demographic, physiological, and biochemical. AI-based data analytics can integrate these multidimensional data sources to provide a more comprehensive assessment and improve early prediction of scoliosis progression. Several studies emphasized the use of mobile apps and IoT-based devices in delivering real-time feedback to improve brace adherence, posture correction, and ROM assessment [72,74,76]. AI-assisted applications and cloud-based systems can improve remote control of orthopedic interventions. In addition, the use of immersive technologies like VR and AR has expanded in recent years. These tools enabled gamified and interactive rehabilitation, often leading to better adherence and physiological improvements in comparison to conventional methods. 

### 4.2. Limitations of Existing Evidence

Despite significant progress in the last five years in the brace treatment for AIS, several limitations remain. Lack of clinical validation was apparent in some studies, especially those involving prototypes or simulations. A few studies validated their methods using either a single participant or none (N = 0 or 1). Sensor-related challenges limited their application as a biomedical device in clinical settings despite showing promising results in the laboratory environment. Issues such as calibration complexity, sensor drift, and difficulties in data interpretation were commonly noted. Limited long-term evidence on the wearable device, along with concerns about data quality and reliability, posed significant limitations in some studies. The durability, wearability, and sustained clinical performance of 3D-printed and smart braces were insufficiently examined in most studies.

AI-based approaches have demonstrated potential in advancing scoliosis care. However, several critical gaps remain that, if addressed, could significantly enhance clinical outcomes. These include the need for large, diverse datasets to train robust models, ensuring user privacy, and navigating regulatory pathways for AI-powered medical devices. Moreover, successful integration into clinical workflows and building trust among practitioners will be essential for widespread adoption. 

Robotic exoskeletons and spine correction devices can apply complex forces on the torso to move the spine to normal posture. Studies showed their successful application in controlling spine posture and accurate torso deformation measurements [7,80]. These devices can address the passive design of braces and lack of adaptive changes in the toros throughout treatment; however, their clinical implementation remains limited by the size and price of these technologies. Ongoing advancements in control systems and manufacturing techniques envision a highlighted role of these technologies in scoliosis treatment. Furthermore, combining AI may allow fully customized, data-driven exoskeletons to be fabricated rapidly and cost-effectively.

In the field of digital and remote therapeutic platforms, there are other limitations such as variability in the use of platforms, accuracy of sensors, and algorithmic reliability, which may lead to complications in standardization and real-world integration. In addition, Issues related to data privacy, security, and regulatory approval remain unresolved in many cases. Another limitation of this scoping review is the exclusion of patents and conference proceedings, which, despite offering valuable insights into emerging technologies, were omitted to maintain methodological consistency and focus on peer-reviewed literature. Future research could address this gap to provide a more comprehensive view of academic and industrial innovations.

### 4.3. Future Directions

The integration of 3D printing, real-time sensor feedback, and adaptive actuation in hybrid orthoses presents a promising path toward improving the non-surgical management of scoliosis.

HDT is a recent concept that has shown growing applications, specifically accelerated through the application of Internet of Medical Things (IoMT). HDT in scoliosis treatment is a virtual representation of patient spines that can simulate and optimize brace design in real time. An early use of the HDT in monitoring patients with scoliosis was introduced by Yates et al. [77], where commercially available smartwatches were used to predict delayed recovery and early onset of postoperative complications. Looking forward, multi-modal AI systems are expected to integrate wearable sensor data, imaging, and patient-reported outcomes to drive digital twin models. In parallel, incorporating additional clinical parameters, such as age, sex, growth rate, or measures of skeletal maturity, may improve the prediction accuracy of AI methods. Further clinical validation will facilitate the application of predictive models to support objective clinical decisions using the AI methods. 

As brace treatment for scoliosis care continues to evolve by integrating technologies and personalized treatment approaches, some areas to prioritize in future research were identified by the authors. Large-scale, multicenter clinical trials may address the concern about long-term safety, reliability, effectiveness, and usability of smart and 3D-printed orthoses. To improve the consistency of outcome assessment for innovative brace treatments, standardized measures, such as objective adherence data, quality of life scores, and functional performance indicators, should be established. This would enable more robust comparisons across different technologies. It seems that a relatively small portion of innovative solutions that showed promising potential to improve brace treatment have been translated into clinical practice. Interdisciplinary collaboration among engineers, orthotists, and clinicians could accelerate the translation of lab-based innovations into routine clinical practice. Finally, ethical considerations such as data privacy, pediatric usability, and equitable access must be incorporated into the design and deployment of next-generation orthoses.

## 5. Conclusions

This scoping review highlighted the advancements in wearable technologies for the treatment of scoliosis between 2020 and 2025, with an emphasis on their role in personalized and non-invasive care. A large portion of studies focused on sensor-integrated orthotic technologies, including smart braces, IMU-based systems, and 3D-printed solutions, which led to enhancing comfort, adherence, and biomechanical correction. Studies showed that EMG-based wearables can facilitate early detection and neuromuscular monitoring, while digital and remote therapeutic platforms have led to enhanced accessibility and more engagement in therapy and treatment. The integration of AI and ML in scoliosis treatment is an emerging research area requiring further validation to be used in predictive analytics, personalized brace design, and remote monitoring.

## Supplementary Materials

The following supporting information can be downloaded at: https://www.mdpi.com/article/10.3390/bioengineering12070696/s1, Table S1: PRISMA 2020 Checklist. Table S2: Studies Excluded After Full-Text Screening with Justifications. Refs. [89,90,91,92,93,94,95,96,97,98,99,100,101,102,103,104,105,106,107,108,109,110,111,112,113,114,115,116,117,118,119,120,121,122,123,124,125,126,127,128,129,130,131,132,133,134,135,136,137,138,139,140,141,142,143,144,145,146,147,148,149,150,151,152,153,154,155,156,157,158,159,160,161,162,163,164,165,166,167,168,169,170,171,172,173,174,175,176,177,178,179,180,181,182,183,184,185,186,187,188,189,190,191,192,193,194,195,196,197,198,199,200,201,202,203,204,205,206,207,208,209,210,211,212,213,214,215,216,217,218,219,220,221,222,223,224,225,226,227,228,229,230,231,232,233,234,235,236,237,238,239,240,241,242,243,244,245,246,247,248,249,250,251,252,253,254,255,256,257].

## Abbreviations

AIS	Adolescent idiopathic scoliosis
ASD	Adult spinal deformity
AI	Artificial intelligence
ATR	Axial trunk rotation
BPMK	Body pressure mapping knitwear
CO	Conventional orthosis
DSC	Dynamic segmental calibration
EMG	Electromyography
EOS	Early-onset scoliosis
FBG	Fiber Bragg grating
FEM	Finite element method
FEA	Finite element analysis
HDT	Human digital twin
IPDs	Implanted programmable devices
IVR	Immersive virtual reality
IMU	Inertial measurement unit
ML	Machine learning
MCGRs	Magnetically controlled growing rods
MB-FE	Multi-body finite element
PETG	Polyethylene terephthalate glycol-modified
PSSE	Physiotherapeutic scoliosis-specific exercises
PACS	Picture archiving and communication system
PCG	Posture correction girdle
PO	Pressure-adjustable orthosis
RMSE	Root mean square error
ROM	Range of motion
SVM	Support vector machine
TLSO	Thoracolumbosacral orthoses
VR	Virtual reality
