# Supplementary material for: Wearable Devices in Scoliosis Treatment: A Scoping Review of Innovations and Challenges
Source: Bioengineering (Basel). 2025 Jun 25;12(7):696. doi: 10.3390/bioengineering12070696 (PMC12292784; doi:10.3390/bioengineering12070696)
Supplement: Supplementary file 1 [file bioengineering-12-00696-s001.zip › bioengineering-3672903-supplementary.pdf]

Supplementary Material
Wearable Devices in Scoliosis Treatment: A Scoping Review of Innovations and Challenges

Samira Fazeli Veisari , Shahrbanoo Bidari , Kourosh Barati, Rasha Atlasi, and Amin Komeili

This supplementary file provides transparent and in-depth details of the search strategy used to identify and collate published evidence on wearable devices in scoliosis treatment. It reports, in full, the exact queries, databases, date ranges, and controlled vocabulary terms employed during the systematic literature search that underpins our review.
In Table S1, the PRISMA 2020 Checklist is presented, and in Table S2, the list studies and the reason for excluding them from the review were provided, following with the bibliography.

Search date: 29 March 2025

Pubmed:

("Wearable Electronic Devices"[Mesh] OR Wearable*[TIAB] OR (Electronic [TIAB] AND Skin[TIAB]) OR "Fitness Trackers"[Mesh] OR (Fitness[TIAB] AND Tracker*[TIAB]) OR (Activity[TIAB] AND Tracker*[TIAB]) OR "Hearing Aids"[Mesh] OR (Hearing [TIAB] AND Aid[TIAB]) OR (Hearing [TIAB] AND Aids[TIAB]) OR "Smart Glasses"[Mesh] OR (Glass*[TIAB] AND Smart[TIAB]) OR Smartglass*[TIAB] OR (“Head Up” [TIAB] AND Display*[TIAB]) OR (Head [TIAB] AND Mounted [TIAB] AND Display*[TIAB]) OR (Head [TIAB] AND Worn [TIAB] AND Display*[TIAB]) OR (Google[TIAB] AND Glass*[TIAB]) OR (Body-worn[TIAB] AND device*[TIAB]) OR (Portable [TIAB] AND smart[TIAB] AND device*[TIAB]) OR (On-body[TIAB] AND device*[TIAB]) OR (Body-mounted[TIAB] AND device*[TIAB]) OR (Smart [TIAB] AND body[TIAB] AND accessor*[TIAB]) OR (Smart[TIAB] AND clothing[TIAB])

OR

(body-borne[TIAB] AND computer*[TIAB])

OR

(head-mounted[TIAB] AND system[TIAB])

OR

(AR [TIAB] AND headset[TIAB])

OR (AR-HUD[TIAB])

OR (ARHMD[TIAB])

OR (augmented [TIAB] AND reality[TIAB] AND glass*[TIAB])

OR (HMD-AR[TIAB])

OR (OST-HMD[TIAB])

OR (see-through[TIAB] AND HMD[TIAB])

OR (head-mounted[TIAB] AND virtual[TIAB] AND reality[TIAB])

OR “OptiVu” [TIAB]

OR (virtual [TIAB] AND reality[TIAB])

OR (Augmented [TIAB] AND reality[TIAB])

OR (VR [TIAB] AND (glass* [TIAB] OR “head set” [TIAB] OR headset[TIAB] OR helmet [TIAB] OR HMD[TIAB]))

OR VR-HMD[TIAB]

OR VRHMD[TIAB]

OR (health[TIAB] AND watch[TIAB])

OR (smart[TIAB] AND watch[TIAB])

OR

(Electronic[TIAB] AND monitoring[TIAB])

OR

Sensor[TIAB]

OR

Sensors[TIAB]

OR

ScoliBrace* [TIAB]

OR

SpineCor[TIAB]

OR

(Smart[TIAB] AND Brace*[TIAB])

OR

(Smart[TIAB] AND Implant*[TIAB])

OR

(Posture[TIAB] AND Monitoring[TIAB])

OR

(UPRIGHT [TIAB] AND GO[TIAB])

OR

(Lumo [TIAB] AND Lift[TIAB])

OR

(Posture [TIAB] AND Tracker*[TIAB])

OR

EMS [TIAB]

OR

(Electrical [TIAB] AND Muscle[TIAB] AND Stimulation[TIAB])

OR

(Neurotech [TIAB] AND ActiGait[TIAB])

OR

(NeuroMD[TIAB] AND Corrective [TIAB] AND Device*[TIAB])

OR

(Smart [TIAB] AND Shirt[TIAB])

OR

VR-Based [TIAB]

OR

(VR[TIAB] AND Therapy[TIAB])

OR

(Posture[TIAB] AND Reminder [TIAB])

OR

EMG [TIAB]

OR

(Noraxon[TIAB] AND MyoTrace[TIAB])

OR

VirtuReal [TIAB]

OR

(Kinect-based[TIAB] AND Posture[TIAB] AND Analysis[TIAB])

OR

(Functional [TIAB] AND Electrical[TIAB] AND Stimulation [TIAB])

OR

FES[TIAB]

OR

(Neurotech [TIAB] AND Functional [TIAB] AND Stimulation[TIAB]))

AND

("Scoliosis"[Mesh] OR Scolioses[TIAB]

OR (Spinal[TIAB] AND curvature[TIAB]) OR (Lateral [TIAB] AND spinal[TIAB] AND deviation[TIAB]) OR (Vertebral [TIAB] AND curvature[TIAB] AND disorder[TIAB]) OR

(Spinal[TIAB] AND deformity[TIAB]) OR (Curved[TIAB] AND spine [TIAB] AND condition[TIAB]))

RESULTS: 374

Web of Science:

S=((Wearable* OR (Electronic AND Skin) OR (Fitness AND Tracker*) OR (Activity AND Tracker*) OR (Hearing AND Aid) OR (Hearing AND Aids) OR (Glass* AND Smart) OR Smartglass* OR ("Head Up" AND Display*) OR (Head AND Mounted AND Display*) OR

(Head AND Worn AND Display*) OR (Google AND Glass*) OR (Body-worn AND device*) OR (Portable AND smart AND device*) OR (On-body AND device*) OR (Body-mounted AND device*) OR (Smart AND body AND accessor*) OR (Smart AND clothing) OR (body-borne AND computer*) OR (head-mounted AND system) OR (AR AND headset) OR (AR-HUD) OR (ARHMD) OR (augmented AND reality AND glass*) OR (HMD-AR) OR (OST-HMD) OR (see-through AND HMD) OR (head-mounted AND virtual AND reality) OR "OptiVu" OR (virtual AND reality) OR (Augmented AND reality) OR (VR AND (glass* OR "head set" OR headset OR helmet OR HMD)) OR VR-HMD OR VRHMD OR (health AND watch) OR (smart AND watch) OR (Electronic AND monitoring) OR Sensor OR Sensors OR ScoliBrace* OR SpineCor OR (Smart AND Brace*) OR (Smart AND Implant*) OR (Posture AND Monitoring) OR (UPRIGHT AND GO) OR (Lumo AND Lift) OR (Posture AND Tracker*) OR EMS OR (Electrical AND Muscle AND Stimulation) OR (Neurotech AND ActiGait) OR (NeuroMD AND Corrective AND Device*) OR (Smart AND Shirt) OR VR-Based OR (VR AND Therapy) OR (Posture AND Reminder) OR EMG OR (Noraxon AND MyoTrace) OR VirtuReal OR (Kinect-based AND Posture AND Analysis) OR (Functional AND Electrical AND Stimulation) OR FES OR (Neurotech AND Functional AND Stimulation))

AND

(Scolioses OR (Spinal AND curvature) OR (Lateral AND spinal AND deviation) OR (Vertebral AND curvature AND disorder) OR (Spinal AND deformity) OR (Curved AND spine AND condition)) **)**

Results: 1013

SCOPUS:

TITLE-ABS-KEY((Wearable* OR (Electronic AND Skin) OR (Fitness AND Tracker*) OR (Activity AND Tracker*) OR (Hearing AND Aid) OR (Hearing AND Aids) OR (Glass* AND Smart) OR Smartglass* OR ("Head Up" AND Display*) OR (Head AND Mounted AND Display*) OR (Head AND Worn AND Display*) OR (Google AND Glass*) OR (Body-worn AND device*) OR (Portable AND smart AND device*) OR (On-body AND device*) OR (Body-mounted AND device*) OR (Smart AND body AND accessor*) OR (Smart AND clothing) OR (body-borne AND computer*) OR (head-mounted AND system) OR (AR AND headset) OR (AR-HUD) OR (ARHMD) OR (augmented AND reality AND glass*) OR (HMD-AR) OR (OST-HMD) OR (see-through AND HMD) OR (head-mounted AND virtual AND reality) OR "OptiVu" OR (virtual AND reality) OR (Augmented AND reality) OR (VR AND (glass* OR "head set" OR headset OR helmet OR HMD)) OR VR-HMD OR VRHMD

OR (health AND watch) OR (smart AND watch) OR (Electronic AND monitoring) OR Sensor OR Sensors OR ScoliBrace* OR SpineCor OR (Smart AND Brace*) OR (Smart AND Implant*) OR (Posture AND Monitoring) OR (UPRIGHT AND GO) OR (Lumo AND Lift) OR (Posture AND Tracker*) OR EMS OR (Electrical AND Muscle AND Stimulation) OR (Neurotech AND ActiGait) OR (NeuroMD AND Corrective AND Device*) OR (Smart AND Shirt) OR VR-Based OR (VR AND Therapy) OR (Posture AND Reminder) OR EMG OR (Noraxon AND MyoTrace) OR VirtuReal OR (Kinect-based AND Posture AND Analysis) OR (Functional AND Electrical AND Stimulation) OR FES OR (Neurotech AND Functional AND Stimulation))

AND

(Scolioses OR (Spinal AND curvature) OR (Lateral AND spinal AND deviation) OR (Vertebral AND curvature AND disorder) OR (Spinal AND deformity) OR (Curved AND spine AND condition)))

Results: 918

EMBASE:

('wearable computer'/exp OR 'activity tracker'/exp OR 'hearing aid'/exp OR 'smart glasses'/exp OR Wearable*:ti,ab,kw OR (Electronic:ti,ab,kw AND Skin:ti,ab,kw) OR (Fitness:ti,ab,kw AND Tracker*:ti,ab,kw) OR (Activity:ti,ab,kw AND Tracker*:ti,ab,kw) OR (Hearing:ti,ab,kw AND Aid:ti,ab,kw) OR (Hearing:ti,ab,kw AND Aids:ti,ab,kw) OR (Glass*:ti,ab,kw AND Smart:ti,ab,kw) OR Smartglass*:ti,ab,kw OR ("Head Up":ti,ab,kw AND Display*:ti,ab,kw) OR (Head:ti,ab,kw AND Mounted:ti,ab,kw AND Display*:ti,ab,kw) OR (Head:ti,ab,kw AND Worn:ti,ab,kw AND Display*:ti,ab,kw) OR (Google:ti,ab,kw AND Glass*:ti,ab,kw) OR (Body-worn:ti,ab,kw AND device*:ti,ab,kw) OR (Portable:ti,ab,kw AND smart:ti,ab,kw AND device*:ti,ab,kw) OR (On-body:ti,ab,kw AND device*:ti,ab,kw) OR (Body-mounted:ti,ab,kw AND device*:ti,ab,kw) OR (Smart:ti,ab,kw AND body:ti,ab,kw AND accessor*:ti,ab,kw) OR (Smart:ti,ab,kw AND clothing:ti,ab,kw)

OR

(body-borne:ti,ab,kw AND computer*:ti,ab,kw)

OR

(head-mounted:ti,ab,kw AND system:ti,ab,kw)

OR

(AR:ti,ab,kw AND headset:ti,ab,kw)

OR (AR-HUD:ti,ab,kw)

OR (ARHMD:ti,ab,kw)

OR (augmented:ti,ab,kw AND reality:ti,ab,kw AND glass*:ti,ab,kw)

OR (HMD-AR:ti,ab,kw)

OR (OST-HMD:ti,ab,kw)

OR (see-through:ti,ab,kw AND HMD:ti,ab,kw)

OR (head-mounted:ti,ab,kw AND virtual:ti,ab,kw AND reality:ti,ab,kw)

OR "OptiVu":ti,ab,kw

OR (virtual:ti,ab,kw AND reality:ti,ab,kw)

OR (Augmented:ti,ab,kw AND reality:ti,ab,kw)

OR (VR:ti,ab,kw AND (glass*:ti,ab,kw OR "head set":ti,ab,kw OR headset:ti,ab,kw OR helmet:ti,ab,kw OR HMD:ti,ab,kw))

OR VR-HMD:ti,ab,kw

OR VRHMD:ti,ab,kw

OR (health:ti,ab,kw AND watch:ti,ab,kw)

OR (smart:ti,ab,kw AND watch:ti,ab,kw)

OR

(Electronic:ti,ab,kw AND monitoring:ti,ab,kw)

OR

Sensor:ti,ab,kw

OR

Sensors:ti,ab,kw

OR

ScoliBrace*:ti,ab,kw

OR

SpineCor:ti,ab,kw

OR

(Smart:ti,ab,kw AND Brace*:ti,ab,kw)

OR

(Smart:ti,ab,kw AND Implant*:ti,ab,kw)

OR

(Posture:ti,ab,kw AND Monitoring:ti,ab,kw)

OR

(UPRIGHT:ti,ab,kw AND GO:ti,ab,kw)

OR

(Lumo:ti,ab,kw AND Lift:ti,ab,kw)

OR

(Posture:ti,ab,kw AND Tracker*:ti,ab,kw)

OR

EMS:ti,ab,kw

OR

(Electrical:ti,ab,kw AND Muscle:ti,ab,kw AND Stimulation:ti,ab,kw)

OR

(Neurotech:ti,ab,kw AND ActiGait:ti,ab,kw)

OR

(NeuroMD:ti,ab,kw AND Corrective:ti,ab,kw AND Device*:ti,ab,kw)

OR

(Smart:ti,ab,kw AND Shirt:ti,ab,kw)

OR

VR-Based:ti,ab,kw

OR

(VR:ti,ab,kw AND Therapy:ti,ab,kw)

OR

(Posture:ti,ab,kw AND Reminder:ti,ab,kw)

OR

EMG:ti,ab,kw

OR

(Noraxon:ti,ab,kw AND MyoTrace:ti,ab,kw)

OR

VirtuReal:ti,ab,kw

OR

(Kinect-based:ti,ab,kw AND Posture:ti,ab,kw AND Analysis:ti,ab,kw)

OR

(Functional:ti,ab,kw AND Electrical:ti,ab,kw AND Stimulation:ti,ab,kw)

OR

FES:ti,ab,kw

OR

(Neurotech:ti,ab,kw AND Functional:ti,ab,kw AND Stimulation:ti,ab,kw))

AND

('scoliosis'/exp OR Scolioses:ti,ab,kw

OR (Spinal:ti,ab,kw AND curvature:ti,ab,kw) OR (Lateral:ti,ab,kw AND spinal:ti,ab,kw AND deviation:ti,ab,kw) OR (Vertebral:ti,ab,kw AND curvature:ti,ab,kw AND disorder:ti,ab,kw) OR

(Spinal:ti,ab,kw AND deformity:ti,ab,kw) OR (Curved:ti,ab,kw AND spine:ti,ab,kw AND condition:ti,ab,kw))

Results: 510

Table S1: PRISMA 2020 Checklist

Section and Topic	Item #	Checklist item	Location where item is reported
**TITLE**			
Title	1	Identify the report as a systematic review.	This is a scoping review paper not systematic review.
**ABSTRACT**			
Abstract	2	See the PRISMA 2020 for Abstracts checklist.	Page 1.
**INTRODUCTION**			
Rationale	3	Describe the rationale for the review in the context of existing knowledge.	Page 2, paragraph 3, line 64- page 2, paragraph 5, line 80, Page 2, paragraph 6, line 89.
Objectives	4	Provide an explicit statement of the objective(s) or question(s) the review addresses.	page 3, paragraph 2, line 98.
**METHODS**			
Eligibility criteria	5	Specify the inclusion and exclusion criteria for the review and how studies were grouped for the syntheses.	page 3, paragraph 6, line 124. page 4, paragraph 2, line 144.
Information sources	6	Specify all databases, registers, websites, organisations, reference lists and other sources searched or consulted to identify studies. Specify the date when each source was last searched or consulted.	page 3, paragraph 4, line 111.
Search strategy	7	Present the full search strategies for all databases, registers and websites, including any filters and limits used.	Supplementary Material 1 file
Selection process	8	Specify the methods used to decide whether a study met the inclusion criteria of the review, including how many reviewers screened each record and each report retrieved, whether they worked independently, and if applicable, details of automation tools used in the process.	page 3, paragraph 7, line 129.
Data collection process	9	Specify the methods used to collect data from reports, including how many reviewers collected data from each report, whether they worked independently, any processes for obtaining or confirming data from study investigators, and if applicable, details of automation tools used in the process.	page 3, paragraph 7, line 129.
Data items	10a	List and define all outcomes for which data were sought. Specify whether all results that were compatible with each outcome domain in each study were sought (e.g. for all measures, time points, analyses), and if not, the methods used to decide which results to collect.	Page 5, paragraph 1, line 161.
	10b	List and define all other variables for which data were sought (e.g. participant and intervention characteristics, funding sources). Describe any assumptions made about any missing or unclear information.	Tables 1-4
Study risk of bias assessment	11	Specify the methods used to assess risk of bias in the included studies, including details of the tool(s) used, how many reviewers assessed each study and whether they worked independently, and if applicable, details of automation tools used in the process.	Not applicable for Scoping review, (mentioned at page 3, paragraph 7, line 129).
Effect measures	12	Specify for each outcome the effect measure(s) (e.g. risk ratio, mean difference) used in the synthesis or presentation of results.	Not applicable for Scoping review (As this review is a scoping review without quantitative synthesis, no statistical effect measures (e.g., risk ratios or mean differences) were applied.)
Synthesis	13a	Describe the processes used to decide which studies were eligible for	page 3, paragraph 5, line

Section and Topic	Item #	Checklist item	Location where item is reported
methods		each synthesis (e.g. tabulating the study intervention characteristics and comparing against the planned groups for each synthesis (item #5)).	119- page 4, paragraph 3, line 157- page 5, paragraph 1, line 161
	13b	Describe any methods required to prepare the data for presentation or synthesis, such as handling of missing summary statistics, or data conversions.	5, paragraph 1, line 167.
	13c	Describe any methods used to tabulate or visually display results of individual studies and syntheses.	page 5, paragraph 1, line 161-fig 2-fig 3-fig 4
	13d	Describe any methods used to synthesize results and provide a rationale for the choice(s). If meta-analysis was performed, describe the model(s), method(s) to identify the presence and extent of statistical heterogeneity, and software package(s) used.	Page 3, paragraph 3, line 106. (meta-analysis was not performed)
	13e	Describe any methods used to explore possible causes of heterogeneity among study results (e.g. subgroup analysis, meta-regression).	Not applicable (As this study is a scoping review with no quantitative synthesis or pooled statistical analysis, no subgroup analysis or meta-regression was conducted to explore heterogeneity.)
	13f	Describe any sensitivity analyses conducted to assess robustness of the synthesized results.	Not applicable (As this study is a scoping review with qualitative thematic synthesis and no quantitative pooling of data, sensitivity analyses were not conducted.)
Reporting bias assessment	14	Describe any methods used to assess risk of bias due to missing results in a synthesis (arising from reporting biases).	Not applicable (As this is a scoping review without quantitative synthesis or meta-analysis, no methods were applied to assess risk of bias due to missing results or reporting bias.)
Certainty assessment	15	Describe any methods used to assess certainty (or confidence) in the body of evidence for an outcome.	Not applicable. (This review is a scoping review and did not assess the certainty or confidence in the body of evidence for specific outcomes.)
**RESULTS**			
Study selection	16a	Describe the results of the search and selection process, from the number of records identified in the search to the number of studies included in the review, ideally using a flow diagram.	page 4, paragraph 2, line 144- Figure 1.
	16b	Cite studies that might appear to meet the inclusion criteria, but which were excluded, and explain why they were excluded.	page 4, paragraph 2, line 151 (Table S1 in the Supplementary Material includes a list of studies that initially appeared to meet inclusion criteria but were excluded during full-text screening, with reasons for exclusion

Section and Topic	Item #	Checklist item	Location where item is reported
			provided.)
Study characteristics	17	Cite each included study and present its characteristics.	Each included study is cited, and its characteristics are presented in detail throughout **Tables 1–4**
Risk of bias in studies	18	Present assessments of risk of bias for each included study.	Page 3, paragraph 7, line 129. (This review is a scoping review and did not include formal risk of bias assessments for each study, as the aim was to provide an overview of the literature rather than evaluate study quality or synthesize effect sizes.)
Results of individual studies	19	For all outcomes, present, for each study: (a) summary statistics for each group (where appropriate) and (b) an effect estimate and its precision (e.g. confidence/credible interval), ideally using structured tables or plots.	Not applicable. (As this review is a scoping review without quantitative comparison between intervention groups, no summary statistics, effect estimates, or confidence intervals were presented.)
Results of syntheses	20a	For each synthesis, briefly summarise the characteristics and risk of bias among contributing studies.	The characteristics of studies included in each conceptual grouping were summarized narratively and tabulated in **Tables 1–4**. However, risk of bias was not assessed, as this is a scoping review and the primary objective of this study was to map the breadth of existing evidence, not to evaluate methodological quality or synthesize effect estimates.
	20b	Present results of all statistical syntheses conducted. If meta-analysis was done, present for each the summary estimate and its precision (e.g. confidence/credible interval) and measures of statistical heterogeneity. If comparing groups, describe the direction of the effect.	Not applicable. (As this study is a scoping review and did not include any statistical synthesis or meta-analysis, no effect estimates, confidence intervals, or heterogeneity measures were reported.)
	20c	Present results of all investigations of possible causes of heterogeneity among study results.	Not applicable. (As this study is a scoping review without statistical synthesis, no investigations of heterogeneity (e.g., subgroup analysis or meta-regression) were conducted.)

Section and Topic	Item #	Checklist item	Location where item is reported
	20d	Present results of all sensitivity analyses conducted to assess the robustness of the synthesized results.	Not applicable. (This study is a scoping review without quantitative synthesis; therefore, sensitivity analyses were not conducted.)
Reporting biases	21	Present assessments of risk of bias due to missing results (arising from reporting biases) for each synthesis assessed.	Page 3, paragraph 7, line 129. (As this study is a scoping review without statistical synthesis or effect size estimation, risk of bias due to missing results (e.g., publication bias) was not assessed.)
Certainty of evidence	22	Present assessments of certainty (or confidence) in the body of evidence for each outcome assessed.	Not applicable. (As this study is a scoping review, it did not assess the certainty or confidence in the body of evidence for individual outcomes.)
**DISCUSSION**			
Discussion	23a	Provide a general interpretation of the results in the context of other evidence.	Page 21, paragraph 1, line 387- Page 21, paragraph 2, line 395- Page 21, paragraph 3, line 402- Page 21, paragraph 4, line 416.
	23b	Discuss any limitations of the evidence included in the review.	Page 22, paragraph 2, line 440- Page 22, paragraph 3, line 450- Page 22, paragraph 4, line 456- Page 22, paragraph 5, line 465.
	23c	Discuss any limitations of the review processes used.	page 3, paragraph 7, line 129- Page 22, paragraph 5, line 469.
	23d	Discuss implications of the results for practice, policy, and future research.	Page 23, paragraph 2, line 491.
**OTHER INFORMATION**			
Registration and protocol	24a	Provide registration information for the review, including register name and registration number, or state that the review was not registered.	This scoping review was not registered in any protocol registry.
	24b	Indicate where the review protocol can be accessed, or state that a protocol was not prepared.	No protocol was prepared for this scoping review.
	24c	Describe and explain any amendments to information provided at registration or in the protocol.	Not applicable. (This review was not registered, and no protocol was prepared; therefore, no amendments were made.)
Support	25	Describe sources of financial or non-financial support for the review, and the role of the funders or sponsors in the review.	Page 23, paragraph 4, line 519 (This project was supported by the Natural Sciences and Engineering Research Council of Canada (NSERC)

Section and Topic	Item #	Checklist item	Location where item is reported
			Discovery Grant [grant number 401610] and the Alberta Innovates Postdoctoral Fellowship program. The funders had no role in the design, analysis, interpretation, or writing of the review.)
Competing interests	26	Declare any competing interests of review authors.	Page 24, paragraph 5, line 540.
Availability of data, code and other materials	27	Report which of the following are publicly available and where they can be found: template data collection forms; data extracted from included studies; data used for all analyses; analytic code; any other materials used in the review.	The materials used in this review, including data extraction forms and synthesized datasets, are not publicly available.

From: Page MJ, McKenzie JE, Bossuyt PM, Boutron I, Hoffmann TC, Mulrow CD, et al. The PRISMA 2020 statement: an updated guideline for reporting systematic reviews. BMJ 2021;372:n71. doi: 10.1136/bmj.n71. This work is licensed under CC BY 4.0. To view a copy of this license, visit https://creativecommons.org/licenses/by/4.0/

Table S2: Studies Excluded After Full-Text Screening with Justifications

Citation	Study Title	Reason for Exclusion
Hannink (2020) [1]	The validity of the kinect sensor for the measurement of sagittal spine curvature against the gold standard lateral spinal radiograph	Related to wearable devices in diagnosis of scoliosis
Hannink et al. (2020) [2]	Measurement of sagittal spine curvature: comparing the Kinect depth camera to the flexicurve and digital inclinometers in a clinical population	
Kokabu (2021) [3]	An algorithm for using deep learning convolutional neural networks with three dimensional depth sensor imaging in scoliosis detection	
Kurzeja (2021) [4]	Selected aspects of using surface topography and scoliometer in screening for scoliotic postural asymmetry in girls	
Li (2021) [5]	Design, reliability, and validity of a portable electronic device based on ergonomics for early screening of adolescent scoliosis	
Shchurova et al. (2021) [6]	Analysis of the degree of involvement of the lower limb muscles in the pathological process in adolescents with idiopathic scoliosis	
Tajdari et al. (2021) [7]	Artificial intelligence data-driven 3D model for AIS	
Tang et al. (2021) [8]	Upper Body Posture Recognition Using Inertial Sensors and Recurrent Neural Networks	
Xu et al. (2021) [9]	Machine-learning-based children's pathological gait classification with low-cost gait-recognition system	
Hong (2022) [10]	Measurement of covered curvature based on a tape of integrated accelerometers	
Huang (2022) [11]	Anatomical prior based vertebra modelling for reappearance of human spines	
Sabri (2022) [12]	Integrated Evolving Spiking Neural Network and Feature Extraction Methods for Scoliosis Classification	
Sabri (2022) [13]	The hybrid feature extraction method for classification of adolescence idiopathic scoliosis using Evolving Spiking Neural Network	
Sikidar (2022) [14]	Classification of mild and severe adolescent idiopathic scoliosis (AIS) from healthy subjects via a supervised learning model based on electromyogram and ground reaction force data during gait	
Shepel and Horoshko (2022) [15]	EFFECTIVENESS OF THE APPLICATION OF KINESIOTAPING IN THE RECOVERY TREATMENT OF IDIOPATHIC SCOLIOSIS, DEGREE 1-2	
Simoneau et al. (2022) [16]	Adolescents with idiopathic scoliosis show decreased intermuscular coherence in lumbar paraspinal muscles: A new pathophysiological perspective	
Villi (2022) [17]	Spine pathologies detections: users' requirements, technological development and first results	

Fan (2023) [18]	Electromyographic Discrepancy in Paravertebral Muscle Activity Predicts Early Curve Progression of Untreated Adolescent Idiopathic Scoliosis	
Tileston et al. (2023) [19]	P96. Postoperative mobility following posterior spinal fusion for adolescent idiopathic scoliosis	
Vutan et al. (2023) [20]	The Use of Accelerometers to Track Changes in Cobb Angles During Scoliosis Rehabilitation Exercises	
Ishikawa (2023) [21]	Prediction of Cobb Angle Using Deep Learning Algorithm with Three-Dimensional Depth Sensor Considering the Influence of Garment in Idiopathic Scoliosis	
Constantinescu et al. (2024) [22]	Scoliotic postural alignment in prepubertal children: somatoscopic analysis of anatomical landmarks and development of a working model to limit spinal changes	
Goto et al. (2024) [23]	Clinical Significance of Pose Estimation Methods Compared with Radiographic Parameters in Adolescent Patients with Idiopathic Scoliosis	
Fan (2024) [24]	An All-in-One Array of Pressure Sensors and sEMG Electrodes for Scoliosis Monitoring	
Shangyu (2024) [25]	Combining artifícial intelligence fór diagnosing adolescent idiopathic scoliosis	
Keil et al. (2024) [26]	Improving Therapy for Children with Scoliosis through Reducing Ionizing Radiation by Using Alternative Imaging Methods-A Study Protocol	
Kim et al. (2024) [27]	Explainable Deep-Learning-Based Gait Analysis of Hip-Knee Cyclogram for the Prediction of Adolescent Idiopathic Scoliosis Progression	
Mohamed et al. (2024) [28]	Effect of adding Schroth physiotherapeutic scoliosis specific exercises to standard care in adolescents with idiopathic scoliosis on posture assessed using surface topography: A secondary analysis of a Randomized Controlled Trial (RCT)	
Vongsirinavarat et al. (2024) [29]	Electromyography of paraspinal muscles during self-corrective positions in adolescent idiopathic scoliosis	
Wang et al. (2024) [30]	Advanced Camera-Based Scoliosis Screening via Deep Learning Detection and Fusion of Trunk, Limb, and Skeleton Features	
Yang et al. (2025) [31]	An automated adolescent idiopathic scoliosis assessment and monitoring model based on back surface	
Mohamed (2025) [32]	Three-dimensional markerless surface topography approach with convolutional neural networks for adolescent idiopathic scoliosis screening	
Rauber et al. (2024) [33]	Predicted vs. measured paraspinal muscle activity in adolescent idiopathic scoliosis patients: EMG validation of optimization-based musculoskeletal simulations	
Ahmed (2020) [34]	An Exoskeleton Based Robotic Device for Providing Rehabilitative Therapies to Human Forearm and Wrist Joints (UWM-FWRR)	Related to wearable devices in scoliosis surgery
Barsotti (2020) [35]	Diagnostic accuracy of perioperative electromyography in the positioning of pedicle screws in adolescent idiopathic scoliosis treatment: a cross-sectional diagnostic study	

Carl (2020) [36]	Spine Surgery Supported by Augmented Reality	
McClendon et al. (2020) [37]	Use of Pheno Room, Augmented Reality, and 3-Rod Technique for 3-Dimensional Correction of Adolescent Idiopathic Scoliosis	
Salci et al. (2020) [38]	Electromyographic Evaluation of Early Stage Results of Exoscopic Microdecompressive Spinal Surgery in Dogs	
Zhao et al. (2020) [39]	Spine morphology measuring instrument based on three-dimensional projection position of the spinous process on body surface: Preliminary application in scoliosis	
Corci (2020) [40]	Novel patient-specific 3D-virtual reality visualisation software (SpectoVR) for the planning of spine surgery: a case series of eight patients	
Edstrom (2020) [41]	A Novel Augmented-Reality-Based Surgical Navigation System for Spine Surgery in a Hybrid Operating Room: Design, Workflow, and Clinical Applications	
Edstrom (2020) [42]	Augmented Reality Surgical Navigation in Spine Surgery to Minimize Staff Radiation Exposure	
Edstrom (2020) [43]	Does Augmented Reality Navigation Increase Pedicle Screw Density Compared to Free-Hand Technique in Deformity Surgery? Single Surgeon Case Series of 44 Patients	
Elmi-Terander (2020) [44]	Augmented reality navigation with intraoperative 3D imaging vs fluoroscopy-assisted free-hand surgery for spine fixation surgery: a matched-control study comparing accuracy	
Feng (2020) [45]	Protocol selecting and technical dilemmas of intraoperative neurophysiological monitoring during corrective procedures for pediatric scoliosis	
Halsey (2020) [46]	Neurophysiological monitoring of spinal cord function during spinal deformity surgery: 2020 SRS neuromonitoring information statement	
Inoue (2020) [47]	Objective evaluation of postoperative changes in real-life activity levels in the postoperative course of lumbar spinal surgery using wearable trackers	
Lohre (2020) [48]	Virtual reality in spinal endoscopy: a paradigm shift in education to support spine surgeons	
Peh et al. (2020) [49]	Accuracy of augmented reality surgical navigation for minimally invasive pedicle screw insertion in the thoracic and lumbar spine with a new tracking device	
Ali et al. (2021) [50]	Emerging Super-specialty of Neurology : Intraoperative Neurophysiological Monitoring (IONM) and Experience in Various Neurosurgeries at a Tertiary Care Hospital in Doha, Qatar	
Kovalev et al. (2021) [51]	Smartphone-assisted augmented reality technology for preoperative planning in spine surgery	
Madrid et al. (2021) [52]	Change in triggered EMG thresholds for thoracic pedicle screws caused by pneumothorax during surgery for adolescent idiopathic scoliosis. Report of two cases	
Shao et al. (2021) [53]	Efficacy and safety for combination of t-EMG with O-arm assisted pedicle screw placement in neurofibromatosis type I scoliosis surgery	

Siemionow et al. (2021) [54]	Autonomous lumbar spine pedicle screw planning using machine learning: A validation study	
Yahanda et al. (2021) [55]	First in-human report of the clinical accuracy of thoracolumbar percutaneous pedicle screw placement using augmented reality guidance	
Polyzoidis et al. (2021) [56]	3D printing in adult and pediatric neurosurgery: The present and the future	
Takahashi et al. (2021) [57]	Validity of the Alarm Point in Intraoperative Neurophysiological Monitoring of the Spinal Cord by the Monitoring Working Group of the Japanese Society for Spine Surgery and Related Research A Prospective Multicenter Cohort Study of 1934 Cases	
Sayari et al. (2021) [58]	Success of Surgical Simulation in Orthopedic Training and Applications in Spine Surgery	
Aoyama et al. (2022) [59]	Augmented Reality Device for Preoperative Marking of Spine Surgery Can Improve the Accuracy of Level Identification	
Mohar (2022) [60]	Fatal Fulminant Fat Embolism Syndrome in Adult Spine Deformity Surgery: A Case Report	
Butler et al. (2022) [61]	216. The arrival of augmented reality in MIS: initial results of use for percutaneous pedicle screw instrumentation	
McMahon et al. (2022) [62]	Does the presence of programmable implanted devices in patients with early onset scoliosis alter typical operative and postoperative practices? A survey of spine surgeons<br><br>Does the presence of programmable implanted devices in patients with early onset scoliosis alter typical operative and postoperative practices? A survey of spine surgeons	
Marx et al. (2022) [63]	Results of a biomechanical pilot study to determine the ROM before and after dorsal correction spondylodesis by means of real-time three-dimensional representation using strain gauges	
Hofler et al. (2022) [64]	Surgery for Adult Deformity Correction	
Claeson et al. (2022) [65]	Power-assisted Pedicle Screw Technique Protects Against Risk of Surgeon Overuse Injury A Comparative Electromyography Study of the Neck and Upper Extremity Muscle Groups in a Simulated Surgical Environment	
Courvoisier et al. (2022) [66]	Virtual Scoliosis Surgery Using a 3D-Printed Model Based on Biplanar Radiographs	
Gadella et al. (2022) [67]	TH-278. Surface versus needle electrodes for recording motor evoked potentials in scoliosis surgery. The NERFACE pilot study	
Ishii et al. (2022) [68]	Navigation-Guided Spinal Fusion: MIS Fusion and Reconstruction in Complex Spine Disease and Deformity	
Jahangiri et al. (2022) [69]	Scoliosis Corrective Surgery With Continuous Intraoperative Neurophysiological Monitoring (IONM)	
Kondylakis et al. (2022) [70]	A Digital Health Intervention for Stress and Anxiety Relief in Perioperative Care: Protocol for a Feasibility Randomized Controlled Trial	

Zarchi et al. (2022) [71]	A practical method for real-time detection of pedicle wall breaching during funneling	
Liu et al. (2022) [72]	Clinical accuracy and initial experience with augmented reality-assisted pedicle screw placement: the first 205 screws	
Mozaffari et al. (2022) [73]	Practical Use of Augmented Reality Modeling to Guide Revision Spine Surgery: An Illustrative Case of Hardware Failure and Overriding Spondyloptosis	
Sommer et al. (2022) [74]	Feasibility of smart glasses in supporting spinal surgical procedures in low- and middle-income countries: experiences from East Africa	
Sumdani et al. (2022) [75]	Utility of Augmented Reality and Virtual Reality in Spine Surgery: A Systematic Review of the Literature	
Atai et al. (2023) [76]	173. Can augmented reality data visualization support more effective intraoperative rod optimization? An in-vitro biomechanical study	
Butler et al. (2023) [77]	Augmented reality in minimally invasive spine surgery: early efficiency and complications of percutaneous pedicle screw instrumentation	
Ma et al. (2023) [78]	Personalized Modeling and Analysis of Mild Adolescent Idiopathic Scoliosis Based on OpenSim	
Shetty and Raja (2023) [79]	Current trends and advancements in spine surgery	
Cardozo et al. (2023) [80]	A360: Augmented reality-assisted percutaneous pedicle screw insertion in the cervical vertebrae: A cadaveric proof-of concept study	
Sun et al. (2023) [81]	The application value of 3D finite element analysis technology and 3D printing biological model technology in the precise surgery of degenerative lumbar scoliosis	
Tillman et al. (2023) [82]	An in vivo analysis of implanted programmable device interference during magnetically controlled growing rod lengthenings: a story of 129 lengthenings	
Daroszewski et al. (2023) [83]	Comparison of Motor Evoked Potentials Neuromonitoring Following Pre- and Postoperative Transcranial Magnetic Stimulation and Intraoperative Electrical Stimulation in Patients Undergoing Surgical Correction of Idiopathic Scoliosis	
Garg et al. (2023) [84]	Improvisation in Spinal Surgery Using AR (Augmented Reality), MR (Mixed Reality), and VR (Virtual Reality) †	
Pahwa et al. (2023) [85]	Assessing the Accuracy of Spinal Instrumentation Using Augmented Reality (AR): A Systematic Review of the Literature and Meta-Analysis	
Yahara et al. (2023) [86]	Asymmetric Load Transmission Induces Facet Joint Subchondral Sclerosis and Hypertrophy in Patients with Idiopathic Adolescent Scoliosis: Evaluation Using Finite Element Model and Surgical Specimen	
Sakai et al. (2023) [87]	Adolescent Idiopathic Scoliotic Deformity Correction Surgery Assisted by Smart Glasses Can Enhance Correction Outcomes and Accuracy and Also Improve Surgeon Fatigue	

Alvi et al. (2024) [88]	Accuracy of Intraoperative Neuromonitoring in the Diagnosis of Intraoperative Neurological Decline in the Setting of Spinal Surgery-A Systematic Review and Meta-Analysis	
Bcharah et al. (2024) [89]	Innovations in Spine Surgery: A Narrative Review of Current Integrative Technologies	
Bhimreddy et al. (2024) [90]	Computational Modeling, Augmented Reality, and Artificial Intelligence in Spine Surgery	
Daroszewski et al. (2024) [91]	"Real-Time Neuromonitoring" Increases the Safety and Non-Invasiveness and Shortens the Duration of Idiopathic Scoliosis Surgery	
De Jesus Encarnacion Ramirez et al. (2024) [92]	Integrating Augmented Reality in Spine Surgery: Redefining Precision with New Technologies	
Dietz et al. (2024) [93]	Scoping review of robotics technology in spinal surgery with highlights of the Annual Seattle Science Foundation Course	
Giraldo et al. (2024) [94]	Advances in neurosurgical education: literature review of mixed-reality simulation models and novel mixed-reality spine prototype	
Hiranaka et al. (2024) [95]	The Utility and Feasibility of Smart Glasses in Spine Surgery: Minimizing Radiation Exposure During Percutaneous Pedicle Screw Insertion	
Kanno et al. (2024) [96]	A Novel Intraoperative CT Navigation System for Spinal Fusion Surgery in Lumbar Degenerative Disease: Accuracy and Safety of Pedicle Screw Placement	
Nguyen et al. (2024) [97]	THE ACCURACY OF PULSE-TRAIN STIMULATION FOR IMPLANTATION OF PEDICLE SCREWS IN ADOLESCENT SCOLIOSIS	
Ohashi et al. (2024) [98]	Mixed Reality-Based Navigation for Pedicle Screw Placement: A Preliminary Study Using a 3D-Printed Spine Model	
Saghbiny et al. (2024) [99]	Breach Detection in Spine Surgery Based on Cutting Torque	
Turlip et al. (2024) [100]	Redefining precision: the current and future roles of artificial intelligence in spine surgery	
Winkler et al. (2024) [101]	Mixed reality for spine surgery: a step into the future with a human cadaveric accuracy study	
Leblanc et al. (2024) [102]	Automatic Spinal Canal Breach Detection During Pedicle Screw Placement	
Tanaka et al. (2024) [103]	Low Radiation Protocol for Intraoperative Robotic C-Arm Can Enhance Adolescent Idiopathic Scoliosis Deformity Correction Accuracy and Safety	
Lewis et al. (2024) [104]	Is a 50% loss in IONM signal the appropriate cut-off in spinal deformity surgery?	
Misterska et al. (2024) [105]	Assessing the Efficacy of Cognitive-Behavioral Therapy on Body Image in Adolescent Scoliosis Patients Using Virtual Reality	
Youssef et al. (2024) [106]	Accuracy of augmented reality-assisted pedicle screw placement: a systematic review	

Azad et al. (2025) [107]	Augmented Reality Versus Freehand Spinopelvic Fixation in Spinal Deformity: A Case-Control Study	
Dada et al. (2025) [108]	Evolution of the Minimally Invasive Surgery Transforaminal Lumbar Interbody Fusion: Where Are We Now?	
Giraldo et al. (2025) [109]	Advances in neurosurgical education: literature review of mixed-reality simulation models and novel mixed-reality spine prototype	
Ikwuegbuenyi et al. (2025) [110]	Strategies for Optimizing Clinical Outcomes in Minimally Invasive Spine Surgery	
Kim et al. (2020) [111]	Flat-Feet Prediction Based on a Designed Wearable Sensing Shoe and a PCA-Based Deep Neural Network Model	Not related to scoliosis
Oppelt et al. (2020) [112]	Movement Analysis in Orthopedics and Trauma Surgery - Measurement Systems and Clinical Applications	
Slater et al. (2020) [113]	Evaluation of Digital Technologies Tailored to Support Young People's Self-Management of Musculoskeletal Pain: Mixed Methods Study	
Taslimipour et al. (2020) [114]	Effects of a Virtual Reality Dance Training Program on Kyphosis Angle and Respiratory Parameters in Young Women With Postural Hyperkyphosis: A Randomized Controlled Clinical Trial	
Zaltieri et al. (2020) [115]	A Wearable Device Based on a Fiber Bragg Grating Sensor for Low Back Movements Monitoring	
Ali et al. (2021) [116]	Spinal Deformities and Advancement in Corrective Orthoses	
Javaid and Haleem (2020) [117]	Significant advancements of 4D printing in the field of orthopaedics	
Jung et al. (2021) [118]	Effectiveness of Combined Stretching and Strengthening Exercise Using Rehabilitation Exercise System with a Linear Actuator and MR Damper on Static and Dynamic Sitting Postural Balance: A Feasibility Study	
Yan et al. (2021) [119]	A ToF-based system for recording human sagittal back shape	
Willwacher et al. (2021) [120]	Dorsal muscle fatigue increases thoracic spine curvature in all-out recreational ergometer rowing	
Shi et al. (2021) [121]	Textile-Based Capacitive Pressure Distribution Measurement System for Human Sitting Posture Monitoring	
Asadullah et al. (2021) [122]	Design of a fluid-driven 3D printed spinal posture corrector	
Odeh et al. (2021) [123]	In-vitro 3D Analysis of Sacroiliac Joint Kinematics: Primary and Coupled Motions	
Adibatti et al. (2022) [124]	Survey of Advances in Cobb Angle Measurement for Automatic Spine Detection in X-Ray	
Willemsen et al. (2022) [125]	Vital Role of In-House 3D Lab to Create Unprecedented Solutions for Challenges in Spinal Surgery, Practical Guidelines and Clinical Case Series	
Wu et al. (2022) [126]	Spinal Posture Recognition Device Using Cloud Storage and BP Neural Network Approach Based on Surface Electromyographic Signal	

Asadullah et al. (2022) [127]	Development of an Automatic Air-Driven 3D-Printed Spinal Posture Corrector	
Aydın et al. (2022) [128]	The Influence of Harmonics Filtering for Weak EMG Analysis	
Fan et al. (2022) [129]	A deep learning based 2-dimensional hip pressure signals analysis method for sitting posture recognition	
Hannink et al. (2022) [130]	Validity of sagittal thoracolumbar curvature measurement using a non-radiographic surface topography method	
Lee et al. (2022) [131]	A Spine Assistive Robot With a Routed Twisted String Actuator and a Flat-Back Alleviation Mechanism for Lumbar-Degenerative Flat Back	
Ekambaram, D. and V. Ponnusamy (2023) [132]	AI-assisted Physical Therapy for Post-injury Rehabilitation: Current State of the Art	
Kurland et al. (2023) [133]	A bibliometric analysis of patient-reported outcome measures in adult spinal deformity, and the future of patient-centric outcome assessments in the era of predictive analytics	
Yan and Wang (2023) [134]	iGuard: An Intelligent Sitting Posture Monitoring System with Pressure Sensors	
Casey et al. (2024) [135]	Standing Balance Conditions and Digital Sway Measures for Clinical Trials of Friedreich's Ataxia	
Ding et al. (2024) [136]	Reconstruction of a Human Spine Curve in the Sagittal Plane From the Measured Contour of the Human Back	
Ding et al. (2024) [137]	Subject-Specific Parametric Identification of a Spine-Equivalent Beam Model for Lumbar Force Prediction in Sagittal Plane	
García-Luna et al. (2024) [138]	Concurrent Validity of the Ergotex Device for Measuring Low Back Posture	
Gupta et al. (2024) [139]	Intraoperative neuromonitoring predicts postoperative deficits in severe pediatric spinal deformity patients	
Hou et al. (2024) [140]	Research status and development trend of smart sitting posture correction garment	
Kim et al. (2024) [141]	Comparison of Neck Pain and Posture with Spine Angle Tracking System between Static and Dynamic Computer Monitor Use	
Kovalenko et al. (2024) [142]	Methodology of Botulinum Therapy in the Treatment of Dystonic Scoliosis in Generalized Dystonia (clinical case)	
Leoncini et al. (2024) [143]	24-h continuous non-invasive multiparameter home monitoring of vitals in patients with Rett syndrome by an innovative wearable technology: evidence of an overlooked chronic fatigue status	
Shaheen et al. (2024) [144]	IoT-Based Solution for Detecting and Monitoring Upper Crossed Syndrome	
Shigematsu et al. (2024) [145]	Current trends in intraoperative neurophysiological monitoring among Asia-Pacific countries: an Asia-Pacific Spine Society survey	
Vos et al. (2024) [146]	Evaluation of 100 Dutch cases with 16p11.2 deletion and duplication syndromes; from clinical manifestations towards personalized treatment options	

Yang et al. (2024) [147]	A New Method for Predicting the Porosity of an Interbody Fusion Cage by the Equivalent Material Method	
Girdler et al. (2020) [148]	Emerging Techniques in Diagnostic Imaging for Idiopathic Scoliosis in Children and Adolescents: A Review of the Literature	Review Papers
Ali et al. (2020) [149]	Spinal Deformities and Advancement in Corrective Orthoses	
Huang et al. (2020) [150]	Augmented reality navigation in spine surgery	
Applebaum et al. (2020) [151]	Evaluating the role of surface topography in the surveillance of scoliosis	
Moreira et al. (2020) [152]	Mobile Applications for Assessing Human Posture: A Systematic Literature Review	
Oppelt et al. (2020) [153]	Movement Analysis in Orthopedics and Trauma Surgery - Measurement Systems and Clinical Applications	
Girdler et al. (2020) [148]	Emerging Techniques in Diagnostic Imaging for Idiopathic Scoliosis in Children and Adolescents: A Review of the Literature	
Yan and Vassar (2021) [154]	Neuromuscular electrical stimulation for motor recovery in pediatric neurological conditions: a scoping review	
Kamalapathy et al. (2022) [155]	Artificial Intelligence in Adult Spinal Deformity	
Li et al. (2022) [156]	Which interventions may improve bracing compliance in adolescent idiopathic scoliosis? A systematic review and meta-analysis	
Ameta et al. (2022) [157]	Critical appraisal and systematic review of 3D & 4D printing in sustainable and environment-friendly smart manufacturing technologies	
Syamlan et al. (2022) [158]	Haptic/virtual reality orthopedic surgical simulators: a literature review	
Karandikar et al. (2022) [159]	Machine Learning Applications of Surgical Imaging for the Diagnosis and Treatment of Spine Disorders: Current State of the Art	
Ng et al. (2022) [160]	Is spinal neuromuscular function asymmetrical in adolescents with idiopathic scoliosis compared to those without scoliosis?: A narrative review of surface EMG studies	
Grivas et al. (2022) [161]	Nonoperative management of adolescent idiopathic scoliosis (AIS) using braces	
Webster (2022) [162]	25 years of commercializing nanomedicine: from tissue engineering to fighting COVID	
Bottino et al. (2023) [163]	Distributed ICT solutions for scoliosis management	
Cerillo et al. (2023) [164]	Spine Bracing: When to Utilize-A Narrative Review	
Cordani et al. (2023) [165]	Influence of Specific Interventions on Bracing Compliance in Adolescents with Idiopathic Scoliosis-A Systematic Review of Papers Including Sensors' Monitoring	
Kurland et al. (2023) [166]	P464: Smartphones, wearables, and digital biomarkers: The evolution of spine care outcomes research	

Khan et al. (2024) [167]	Electrical Stimulation Methods for Scoliosis in Children: a Literature Review	
Paramento et al. (2024) [168]	Neurophysiological, balance and motion evidence in adolescent idiopathic scoliosis: A systematic review	
Kim, H., et al. (2024) [169]	Current issues in the treatment of adolescent idiopathic scoliosis: a comprehensive narrative review	
Cheng et al. (2024) [170]	Non-invasive Scoliosis Assessment in Adolescents	
Agravante et al. (2024) [171]	Emerging Technologies of Sensor-Based Assistive Devices for Spinal Position Monitoring: A Review	
Alvi et al. (2024) [172]	Accuracy of Intraoperative Neuromonitoring in the Diagnosis of Intraoperative Neurological Decline in the Setting of Spinal Surgery—A Systematic Review and Meta-Analysis	
Lee et al. (2024) [173]	Artificial Intelligence in Spinal Imaging and Patient Care: A Review of Recent Advances	
Karimi and Kavyani (2024) [174]	Evaluation of the effectiveness of soft braces on idiopathic scoliosis: A review of literature	
Förstl et al. (2024) [175]	Technologies for Evaluation of Pelvic Floor Functionality: A Systematic Review	
Ratnaparkhi and Beckett (2024) [176]	Digital Phenotyping, Wearables, and Outcomes	
Luo et al. (2024) [177]	The Biomechanics of Spinal Orthoses for Adolescent Idiopathic Scoliosis: A Systematic Review of the Controlling Forces	
Kaelin (2020) [178]	Adolescent idiopathic scoliosis: indications for bracing and conservative treatments	Other reasons
Tapp et al. (2021) [179]	Generation of Patient-Specific, Ligamentoskeletal, Finite Element Meshes for Scoliosis Correction Planning	
Negrini et al. (2022) [180]	The classification of scoliosis braces developed by SOSORT with SRS, ISPO, and POSNA and approved by ESPRM	
Te Hennepe et al. (2025) [181]	Pulmonary function in patients with adolescent idiopathic scoliosis: an explorative study of a wearable smart shirt as a measurement instrument	

Bibliography:

1. Hannink, E.; Shannon, T.; Dawes, H.; Barker, K. The validity of the kinect sensor for the measurement of sagittal spine curvature against the gold standard lateral spinal radiograph. *Physiotherapy (United Kingdom)* **2020**, *107*, e28-e29, doi:10.1016/j.physio.2020.03.040.
2. Hannink, E.; Shannon, T.; Dawes, H.; Barker, K. Measurement of sagittal spine curvature: comparing the Kinect depth camera to the flexicurve and digital inclinometers in a clinical population. *Physiotherapy (United Kingdom)* **2020**, *107*, e21, doi:10.1016/j.physio.2020.03.031.
3. Kokabu, T.; Kanai, S.; Kawakami, N.; Uno, K.; Kotani, T.; Suzuki, T.; Tachi, H.; Abe, Y.; Iwasaki, N.; Sudo, H. An algorithm for using deep learning convolutional neural networks with three dimensional depth sensor imaging in scoliosis detection. *Spine Journal* **2021**, *21*, 980-987, doi:10.1016/j.spinee.2021.01.022.
4. Kurzeja, P.; Ogrodzka-Ciechanowicz, K.; Rozek, K.; Hudakova, Z. Selected aspects of using surface topography and scoliometer in screening for scoliotic postural asymmetry in girls. *Archives of Budo Science of Martial Arts and Extreme Sports* **2021**, *17*, 29-39.
5. Li, C.; Zhang, B.; Liu, L.; Li, Y.; Xu, Y.; Wang, L.; Yun, C.; Zhao, Y. Design, reliability, and validity of a portable electronic device based on ergonomics for early screening of adolescent scoliosis. *Journal of Orthopaedic Translation* **2021**, *28*, 83-89, doi:10.1016/j.jot.2020.10.014.
6. Shchurova, E.N.; Saifutdinov, M.S.; Dolganova, T.I.; Akhmedova, M.A.; Gorbach, A.P. Analysis of the degree of involvement of the lower limb muscles in the pathological process in adolescents with idiopathic scoliosis. *Acta Biomedica Scientifica* **2021**, *6*, 154-165, doi:10.29413/ABS.2021-6.2.18.
7. Tajdari, M.; Maqsood, A.; Li, H.; Saha, S.; Sarwark, J.F.; Liu, W.K. Artificial intelligence data-driven 3D model for AIS. In Proceedings of the Studies in Health Technology and Informatics, 2021; pp. 141-145.
8. Tang, H.Y.; Tan, S.H.; Su, T.Y.; Chiang, C.J.; Chen, H.H. Upper Body Posture Recognition Using Inertial Sensors and Recurrent Neural Networks. *Applied Sciences-Basel* **2021**, *11*, doi:10.3390/app112412101.
9. Xu, L.H.; Chen, J.S.; Wang, F.; Chen, Y.T.; Yang, W.; Yang, C.J. Machine-learning-based children's pathological gait classification with low-cost gait-recognition system. *Biomedical Engineering Online* **2021**, *20*, doi:10.1186/s12938-021-00898-0.
10. Hong, T.T.H.; Wang, Y.; Tan, Q.; Zhang, G.; Wong, D.W.C.; Zhang, M. Measurement of covered curvature based on a tape of integrated accelerometers. *Measurement: Journal of the International Measurement Confederation* **2022**, *193 C7 - 110959*, doi:10.1016/j.measurement.2022.110959.
11. Huang, Q.; Luo, H.; Yang, C.; Li, J.; Deng, Q.; Liu, P.; Fu, M.; Li, L.; Li, X. Anatomical prior based vertebra modelling for reappearance of human spines. *Neurocomputing* **2022**, *500*, 750-760, doi:10.1016/j.neucom.2022.05.033.

12. Sabri, N.; Hamed, H.N.A.; Ibrahim, Z.; Ibrahim, K.; Isa, M.A. Integrated Evolving Spiking Neural Network and Feature Extraction Methods for Scoliosis Classification. *Computers, Materials and Continua* **2022**, *73*, 5559-5573, doi:10.32604/cmc.2022.029221.
13. Sabri, N.; Hamed, H.N.A.; Ibrahim, Z.; Ibrahim, K.; Isa, M.A.; Diah, N.M. The hybrid feature extraction method for classification of adolescence idiopathic scoliosis using Evolving Spiking Neural Network. *Journal of King Saud University - Computer and Information Sciences* **2022**, *34*, 8899-8908, doi:10.1016/j.jksuci.2022.08.019.
14. Sikidar, A.; Vidyasagar, K.E.C.; Gupta, M.; Garg, B.; Kalyanasundaram, D. Classification of mild and severe adolescent idiopathic scoliosis (AIS) from healthy subjects via a supervised learning model based on electromyogram and ground reaction force data during gait. *Biocybernetics and Biomedical Engineering* **2022**, *42*, 870-887, doi:10.1016/j.bbe.2022.06.006.
15. Shepel, A.I.; Horoshko, V.I. EFFECTIVENESS OF THE APPLICATION OF KINESIOTAPING IN THE RECOVERY TREATMENT OF IDIOPATHIC SCOLIOSIS, DEGREE 1-2. *Rehabilitation and Recreation* **2022**, *2022*, 81-85, doi:10.32782/2522-1795.2022.13.10.
16. Simoneau, M.; Pialasse, J.P.; Mercier, P.; Blouin, J.S. Adolescents with idiopathic scoliosis show decreased intermuscular coherence in lumbar paraspinal muscles: A new pathophysiological perspective. *Clinical Neurophysiology* **2022**, *138*, 38-51, doi:10.1016/j.clinph.2022.03.008.
17. Villi, H.; Pinsault, N.; Thomann, G. Spine pathologies detections: users' requirements, technological development and first results. In Proceedings of the Procedia CIRP, 2022; pp. 209-214.
18. Fan, Y.; To, M.K.T.; Yeung, E.H.K.; Kuang, G.M.; Liang, R.; Cheung, J.P.Y. Electromyographic Discrepancy in Paravertebral Muscle Activity Predicts Early Curve Progression of Untreated Adolescent Idiopathic Scoliosis. *Asian Spine Journal* **2023**, *17*, 922-932, doi:10.31616/asj.2023.0199.
19. Tileston, K.R.; Naz, K.; Pham, N.; Bryson, X.; Policy, J.; Vorhies, J. P96. Postoperative mobility following posterior spinal fusion for adolescent idiopathic scoliosis. *Spine Journal* **2023**, *23*, S153-S154, doi:10.1016/j.spinee.2023.06.321.
20. Vutan, A.M.; Gruescu, C.M.; Sticlaru, C.; Lovasz, E.C. The Use of Accelerometers to Track Changes in Cobb Angles During Scoliosis Rehabilitation Exercises. *New Trends in Medical and Service Robotics, Mesrob 2023* **2023**, *133*, 279-286, doi:10.1007/978-3-031-32446-8_30.
21. Ishikawa, Y.; Kokabu, T.; Yamada, K.; Abe, Y.; Tachi, H.; Suzuki, H.; Ohnishi, T.; Endo, T.; Ukeba, D.; Ura, K.; et al. Prediction of Cobb Angle Using Deep Learning Algorithm with Three-Dimensional Depth Sensor Considering the Influence of Garment in Idiopathic Scoliosis. *Journal of Clinical Medicine* **2023**, *12*, doi:10.3390/jcm12020499.
22. Constantinescu, M.; Onu, I.; Trofin, D.; Talaghir, L.G.; Coja, D.M.; Iordan, D.A.; Filip, F.; Silisteanu, S.C.; Vizitiu, E.; Musat, C.L.; et al. Scoliotic postural alignment in prepubertal children: somatoscopic analysis of anatomical landmarks and development of a working model to limit spinal changes. *Balneo and Prm Research Journal* **2024**, *15*, doi:10.12680/balneo.2024.655.

23. Goto, G.; Ariga, K.; Tanaka, N.; Oda, K.; Haro, H.; Ohba, T. Clinical Significance of Pose Estimation Methods Compared with Radiographic Parameters in Adolescent Patients with Idiopathic Scoliosis. *Spine Surgery and Related Research* **2024**, *8*, 485-493, doi:10.22603/ssrr.2023-0269.
24. Fan, W.; Wang, S.; Li, Q.; Ren, X.; Zhang, C.; Wang, H.; Li, M.; Yang, W.; Deng, W. An All-in-One Array of Pressure Sensors and sEMG Electrodes for Scoliosis Monitoring. *Small* **2024**, *20*, e2404136, doi:10.1002/smll.202404136.
25. Shangyu, G.; Dahui, W. Combining artifícial intelligence fór diagnosing adolescent idiopathic scoliosis. *Journal of Clinical Pediatric Surgery* **2024**, *23*, 89-92, doi:10.3760/cma.j.cn101785-202211044-018.
26. Keil, F.; Schneider, R.; Polomac, N.; Zabar, O.; Finger, T.; Holzgreve, F.; Czabanka, M.; Erbe, C.; Groneberg, D.A.; Hattingen, E.; et al. Improving Therapy for Children with Scoliosis through Reducing Ionizing Radiation by Using Alternative Imaging Methods-A Study Protocol. *Journal of Clinical Medicine* **2024**, *13*, doi:10.3390/jcm13195768.
27. Kim, Y.G.; Kim, S.; Park, J.H.; Yang, S.; Jang, M.; Yun, Y.J.; Cho, J.S.; You, S.; Jang, S.H. Explainable Deep-Learning-Based Gait Analysis of Hip-Knee Cyclogram for the Prediction of Adolescent Idiopathic Scoliosis Progression. *Sensors (Basel)* **2024**, *24*, doi:10.3390/s24144504.
28. Mohamed, N.; Acharya, V.; Schreiber, S.; Parent, E.C.; Westover, L. Effect of adding Schroth physiotherapeutic scoliosis specific exercises to standard care in adolescents with idiopathic scoliosis on posture assessed using surface topography: A secondary analysis of a Randomized Controlled Trial (RCT). *Plos One* **2024**, *19*, doi:10.1371/journal.pone.0302577.
29. Vongsirinavarat, M.; Kao-Ngampanich, P.; Sinsurin, K. Electromyography of paraspinal muscles during self-corrective positions in adolescent idiopathic scoliosis. *J Back Musculoskelet Rehabil* **2024**, *37*, 165-173, doi:10.3233/bmr-230055.
30. Wang, Z.; Zhou, Y.; Xu, N.; Zhou, Y.; Zhao, H.; Chang, Z.; Hu, Z.; Han, X.; Song, Y.; Zhou, Z.; et al. Advanced Camera-Based Scoliosis Screening via Deep Learning Detection and Fusion of Trunk, Limb, and Skeleton Features. *IEEE Journal of Biomedical and Health Informatics* **2024**, doi:10.1109/JBHI.2024.3491855.
31. Yang, J.; Li, Y.; Hu, A.; Wang, J.; Meng, M.Q.H. An automated adolescent idiopathic scoliosis assessment and monitoring model based on back surface. *Biomedical Signal Processing and Control* **2025**, *102 C7 - 107321*, doi:10.1016/j.bspc.2024.107321.
32. Mohamed, N.; Ruiz, J.M.G.; Hassan, M.; Costa, O.A.; Burke, T.N.; Mei, Q.; Westover, L. Three-dimensional markerless surface topography approach with convolutional neural networks for adolescent idiopathic scoliosis screening. *Sci Rep* **2025**, *15*, 8728, doi:10.1038/s41598-025-92551-2.
33. Rauber, C.; Lüscher, D.; Poux, L.; Schori, M.; Deml, M.C.; Hasler, C.C.; Bassani, T.; Galbusera, F.; Büchler, P.; Schmid, S. Predicted vs. measured paraspinal muscle activity in adolescent idiopathic scoliosis patients: EMG validation of optimization-based musculoskeletal simulations. *Journal of Biomechanics* **2024**, *163*, doi:10.1016/j.jbiomech.2023.111922.

34. Ahmed, T.; Al Zubayer Swapnil, A.; Islam, M.R.; Wang, I.; Rahman, M. An Exoskeleton Based Robotic Device for Providing Rehabilitative Therapies to Human Forearm and Wrist Joints (UWM-FWRR). *Archives of Physical Medicine and Rehabilitation* **2020**, *101*, e56, doi:10.1016/j.apmr.2020.09.167.
35. Barsotti, C.E.; Gavassi, B.M.; Prado, F.E.; Batista, B.N.; de Resende Pratali, R.; Ribeiro, A.P.; de Oliveira, C.E.S.; Ferreira, R.R. Diagnostic accuracy of perioperative electromyography in the positioning of pedicle screws in adolescent idiopathic scoliosis treatment: a cross-sectional diagnostic study. *BMC Musculoskelet Disord* **2020**, *21*, 473, doi:10.1186/s12891-020-03491-z.
36. Carl, B.; Bopp, M.; Sass, B.; Pojskic, M.; Voellger, B.; Nimsky, C. Spine Surgery Supported by Augmented Reality. *Global Spine Journal* **2020**, *10*, 41S-55S, doi:10.1177/2192568219868217.
37. McClendon, J.; Almekkawi, A.K.; Abi-Aad, K.R.; Maiti, T. Use of Pheno Room, Augmented Reality, and 3-Rod Technique for 3-Dimensional Correction of Adolescent Idiopathic Scoliosis. *World Neurosurgery* **2020**, *137*, 291-291, doi:10.1016/j.wneu.2020.01.094.
38. Salci, H.; Acar, H.; Taskapilioglu, M.O. Electromyographic Evaluation of Early Stage Results of Exoscopic Microdecompressive Spinal Surgery in Dogs. *Acta Scientiae Veterinariae* **2020**, *48*, doi:10.22456/1679-9216.101278.
39. Zhao, J.; Chen, K.; Li, Q.h.; Hang, H.d.; Mao, N.f.; Shen, L.y.; Yang, C.w.; Li, M. Spine morphology measuring instrument based on three-dimensional projection position of the spinous process on body surface: Preliminary application in scoliosis. *Academic Journal of Second Military Medical University* **2020**, *41*, 1198-1202, doi:10.16781/j.0258-879x.2020.11.1198.
40. Croci, D.M.; Guzman, R.; Netzer, C.; Mariani, L.; Schaeren, S.; Cattin, P.C.; Jost, G.F. Novel patient-specific 3D-virtual reality visualisation software (SpectoVR) for the planning of spine surgery: a case series of eight patients. *Bmj Innovations* **2020**, *6*, 215-219, doi:10.1136/bmjinnov-2019-000398.
41. Edstrom, E.; Burstrom, G.; Nachabe, R.; Gerdhem, P.; Terander, A.E. A Novel Augmented-Reality-Based Surgical Navigation System for Spine Surgery in a Hybrid Operating Room: Design, Workflow, and Clinical Applications. *Operative Neurosurgery* **2020**, *18*, 496-502, doi:10.1093/ons/opz236.
42. Edström, E.; Burström, G.; Omar, A.; Nachabe, R.; Söderman, M.; Persson, O.; Gerdhem, P.; Elmi-Terander, A. Augmented Reality Surgical Navigation in Spine Surgery to Minimize Staff Radiation Exposure. *Spine* **2020**, *45*, E45-E53, doi:10.1097/BRS.0000000000003197.
43. Edström, E.; Burström, G.; Persson, O.; Charalampidis, A.; Nachabe, R.; Gerdhem, P.; Elmi-Terander, A. Does Augmented Reality Navigation Increase Pedicle Screw Density Compared to Free-Hand Technique in Deformity Surgery? Single Surgeon Case Series of 44 Patients. *Spine* **2020**, *45*, E1085-E1090, doi:10.1097/BRS.0000000000003518.
44. Elmi-Terander, A.; Burström, G.; Nachabé, R.; Fagerlund, M.; Ståhl, F.; Charalampidis, A.; Edström, E.; Gerdhem, P. Augmented reality navigation with intraoperative 3D imaging vs fluoroscopy-assisted free-hand surgery for spine fixation surgery: a

matched-control study comparing accuracy. *Scientific Reports* **2020**, *10*, doi:10.1038/s41598-020-57693-5.
45. Feng, L.; Zhang, X. Protocol selecting and technical dilemmas of intraoperative neurophysiological monitoring during corrective procedures for pediatric scoliosis. *Journal of Clinical Pediatric Surgery* **2020**, *2020*, 93-97, doi:10.3969/j.issn.1671-6353.2020.02.001.
46. Halsey, M.F.; Myung, K.S.; Ghag, A.; Vitale, M.G.; Newton, P.O.; de Kleuver, M. Neurophysiological monitoring of spinal cord function during spinal deformity surgery: 2020 SRS neuromonitoring information statement. *Spine Deformity* **2020**, *8*, 591-596, doi:10.1007/s43390-020-00140-2.
47. Inoue, M.; Orita, S.; Inage, K.; Suzuki, M.; Fujimoto, K.; Shiga, Y.; Kanamoto, H.; Abe, K.; Kinoshita, H.; Norimoto, M.; et al. Objective evaluation of postoperative changes in real-life activity levels in the postoperative course of lumbar spinal surgery using wearable trackers. *BMC Musculoskeletal Disorders* **2020**, *21*, doi:10.1186/s12891-020-3102-2.
48. Lohre, R.; Wang, J.C.; Lewandrowski, K.U.; Goel, D.P. Virtual reality in spinal endoscopy: a paradigm shift in education to support spine surgeons. *J Spine Surg* **2020**, *6*, S208-s223, doi:10.21037/jss.2019.11.16.
49. Peh, S.; Chatterjea, A.; Pfarr, J.; Schäfer, J.P.; Weuster, M.; Klüter, T.; Seekamp, A.; Lippross, S. Accuracy of augmented reality surgical navigation for minimally invasive pedicle screw insertion in the thoracic and lumbar spine with a new tracking device. *Spine Journal* **2020**, *20*, 629-637, doi:10.1016/j.spinee.2019.12.009.
50. Ali, L.; Jahangiri, F.R.; Ali, A.; Belkhair, S.; Elalamy, O.; Adeli, G.; Alghazow, M.; Krishnan, R.; Karim, F.; Iqrar, A.; et al. Emerging Super-specialty of Neurology : Intraoperative Neurophysiological Monitoring (IONM) and Experience in Various Neurosurgeries at a Tertiary Care Hospital in Doha, Qatar. *Cureus Journal of Medical Science* **2021**, *13*, doi:10.7759/cureus.20432.
51. Kovalev, E.V.; Kirilenko, S.I.; Mazurenko, A.N.; Filiustin, A.E.; Dubrovsky, V.V. Smartphone-assisted augmented reality technology for preoperative planning in spine surgery. *Hirurgia Pozvonochnika* **2021**, *18*, 94-99, doi:10.14531/SS2021.3.94-99.
52. Madrid, C.; Gómez-Rice, A.; Cordón, S.; Sanz-Barbero, E.; Zúñiga, L. Change in triggered EMG thresholds for thoracic pedicle screws caused by pneumothorax during surgery for adolescent idiopathic scoliosis. Report of two cases. *Revista Espanola de Cirugia Ortopedica y Traumatologia* **2021**, *65*, 472-476, doi:10.1016/j.recot.2020.09.007.
53. Shao, X.; Huang, Z.; Yang, J.; Deng, Y.; Yang, J.; Sui, W. Efficacy and safety for combination of t-EMG with O-arm assisted pedicle screw placement in neurofibromatosis type I scoliosis surgery. *Journal of Orthopaedic Surgery and Research* **2021**, *16*, doi:10.1186/s13018-021-02882-9.
54. Siemionow, K.B.; Forsthoefel, C.W.; Foy, M.P.; Gawel, D.; Luciano, C.J. Autonomous lumbar spine pedicle screw planning using machine learning: A validation study. *Journal of Craniovertebral Junction and Spine* **2021**, *12*, 223-227, doi:10.4103/jcvjs.jcvjs_94_21.
55. Yahanda, A.T.; Moore, E.; Ray, W.Z.; Pennicooke, B.; Jennings, J.W.; Molina, C.A. First in-human report of the clinical accuracy of thoracolumbar percutaneous pedicle screw

placement using augmented reality guidance. *Neurosurgical Focus* **2021**, *51*, doi:10.3171/2021.5.FOCUS21217.
56. Polyzoidis, S.; McGuire, L.S.; Nikas, D.; Ashkan, K. 3D printing in adult and pediatric neurosurgery: The present and the future. In *3D Printing: Applications in Medicine and Surgery Volume 2*; Elsevier: 2021; pp. 243-259.
57. Takahashi, M.; Imagama, S.; Kobayashi, K.; Yamada, K.; Yoshida, G.; Yamamoto, N.; Ando, M.; Kawabata, S.; Kanchiku, T.; Fujiwara, Y.; et al. Validity of the Alarm Point in Intraoperative Neurophysiological Monitoring of the Spinal Cord by the Monitoring Working Group of the Japanese Society for Spine Surgery and Related Research A Prospective Multicenter Cohort Study of 1934 Cases. *Spine* **2021**, *46*, E1069-E1076, doi:10.1097/BRS.0000000000004065.
58. Sayari, A.J.; Chen, O.; Harada, G.K.; Lopez, G.D. Success of Surgical Simulation in Orthopedic Training and Applications in Spine Surgery. *Clinical Spine Surgery* **2021**, *34*, 82-86, doi:10.1097/BSD.0000000000001070.
59. Aoyama, R.; Anazawa, U.; Hotta, H.; Watanabe, I.; Takahashi, Y.; Matsumoto, S.; Ishibashi, T. Augmented Reality Device for Preoperative Marking of Spine Surgery Can Improve the Accuracy of Level Identification. *Spine Surgery and Related Research* **2022**, *6*, 303-309, doi:10.22603/ssrr.2021-0168.
60. Mohar, J. Fatal Fulminant Fat Embolism Syndrome in Adult Spine Deformity Surgery: A Case Report. *JBJS Case Connect* **2022**, *12*, doi:e22.00181

10.2106/jbjs.Cc.22.00181.
61. Butler, A.J.; Rush, A.J.; Lynch, J.J.; Colman, M.; Phillips, F.M. 216. The arrival of augmented reality in MIS: initial results of use for percutaneous pedicle screw instrumentation. *Spine Journal* **2022**, *22*, S115, doi:10.1016/j.spinee.2022.06.236.
62. McMahon, R.; Morgan, S.J.; Brooks, J.T.; Cahill, P.; Fitzgerald, R.; Li, Y.; Abdullah, A.S.A.; Ahn, E.; Akbarnia, B.; Akoto, H.; et al. Does the presence of programmable implanted devices in patients with early onset scoliosis alter typical operative and postoperative practices? A survey of spine surgeons. *Spine Deformity* **2022**, *10*, 951-964, doi:10.1007/s43390-022-00477-w.
63. Marx, W.; Niemeyer, T.; Conta, A.; Hartmann, C.; Obid, P. Results of a biomechanical pilot study to determine the ROM before and after dorsal correction spondylodesis by means of real-time three-dimensional representation using strain gauges. *European Spine Journal* **2022**, *31*, 3198-3199, doi:10.1007/s00586-022-07413-6.
64. Hofler, R.C.; Dinsmore, T.H.; Fessler, R.G. Surgery for Adult Deformity Correction. In *Koht, Sloan, Toleikis's Monitoring the Nervous System for Anesthesiologists and Other Health Care Professionals: Third Edition*; Springer International Publishing: 2022; pp. 603-616.
65. Claeson, A.A.; Schwab, F.J.; Gandhi, A.A.; Skaggs, D.L. Power-assisted Pedicle Screw Technique Protects Against Risk of Surgeon Overuse Injury A Comparative Electromyography Study of the Neck and Upper Extremity Muscle Groups in a Simulated Surgical Environment. *Spine* **2022**, *47*, E86-E93, doi:10.1097/BRS.0000000000004097.

66. Courvoisier, A.; Cebrian, A.; Simon, J.; Désauté, P.; Aubert, B.; Amabile, C.; Thiébaut, L. Virtual Scoliosis Surgery Using a 3D-Printed Model Based on Biplanar Radiographs. *Bioengineering-Basel* **2022**, *9*, doi:10.3390/bioengineering9090469.
67. Gadella, M.; Dulfer, S.; Lange, F.; Scholtens, C.; Absalom, A.; Groen, R.; Faber, C.; Tamasi, K.; Sahinovic, M.; Drost, G. TH-278. Surface versus needle electrodes for recording motor evoked potentials in scoliosis surgery. The NERFACE pilot study. *Clinical Neurophysiology* **2022**, *141*, S176, doi:10.1016/j.clinph.2022.07.464.
68. Ishii, K.; Isogai, N.; Funao, H. Navigation-Guided Spinal Fusion: MIS Fusion and Reconstruction in Complex Spine Disease and Deformity. In *Technical Advances in Minimally Invasive Spine Surgery: Navigation, Robotics, Endoscopy, Augmented and Virtual Reality*; Springer Nature: 2022; pp. 65-71.
69. Jahangiri, F.R.; Jahangiri, R.H.; Asad, H.; Farooq, L.; Khattak, W.H. Scoliosis Corrective Surgery With Continuous Intraoperative Neurophysiological Monitoring (IONM). *Cureus Journal of Medical Science* **2022**, *14*, doi:10.7759/cureus.29958.
70. Kondylakis, H.; Giglioli, I.A.C.; Katehakis, D.G.; Aldemir, H.; Zikas, P.; Papagiannakis, G.; Hors-Fraile, S.; González-Sanz, P.L.; Apostolakis, K.C.; Stephanidis, C.; et al. A Digital Health Intervention for Stress and Anxiety Relief in Perioperative Care: Protocol for a Feasibility Randomized Controlled Trial. *Jmir Research Protocols* **2022**, *11*, doi:10.2196/38536.
71. Zarchi, O.; Ohana, N.; Mercado, E.; Amitai, A.; Berestizshevsky, Y.; Sheinis, D.; Benharroch, D.; Bar-On, E. A practical method for real-time detection of pedicle wall breaching during funneling. *Archives of Orthopaedic and Trauma Surgery* **2022**, *142*, 3017-3025, doi:10.1007/s00402-021-03885-1.
72. Liu, A.; Jin, Y.; Cottrill, E.; Khan, M.; Westbroek, E.; Ehresman, J.; Pennington, Z.; Lo, S.F.L.; Sciubba, D.M.; Molina, C.A.; et al. Clinical accuracy and initial experience with augmented reality-assisted pedicle screw placement: the first 205 screws. *Journal of Neurosurgery: Spine* **2022**, *36*, 351-357, doi:10.3171/2021.2.SPINE202097.
73. Mozaffari, K.; Foster, C.H.; Rosner, M.K. Practical Use of Augmented Reality Modeling to Guide Revision Spine Surgery: An Illustrative Case of Hardware Failure and Overriding Spondyloptosis. *Operative Neurosurgery* **2022**, *23*, 212-216, doi:10.1227/ons.0000000000000307.
74. Sommer, F.; Waterkeyn, F.; Hussain, I.; Goldberg, J.L.; Kirnaz, S.; Navarro-Ramirez, R.; Ahmad, A.A.; Balsano, M.; Medary, B.; Shabani, H.; et al. Feasibility of smart glasses in supporting spinal surgical procedures in low- and middle-income countries: experiences from East Africa. *Neurosurgical Focus* **2022**, *52*, doi:10.3171/2022.3.FOCUS2237.
75. Sumdani, H.; Aguilar-Salinas, P.; Avila, M.J.; Barber, S.R.; Dumont, T. Utility of Augmented Reality and Virtual Reality in Spine Surgery: A Systematic Review of the Literature. *World Neurosurgery* **2022**, *161*, e8-e17, doi:10.1016/j.wneu.2021.08.002.
76. Atai, N.A.; Mehta, V.; Kobbe, P.; Weidle, P. 173. Can augmented reality data visualization support more effective intraoperative rod optimization? An in-vitro biomechanical study. *Spine Journal* **2023**, *23*, S89, doi:10.1016/j.spinee.2023.06.195.

77. Butler, A.J.; Colman, M.W.; Lynch, J.; Phillips, F.M. Augmented reality in minimally invasive spine surgery: early efficiency and complications of percutaneous pedicle screw instrumentation. *Spine Journal* **2023**, *23*, 27-33, doi:10.1016/j.spinee.2022.09.008.
78. Ma, X.; Ren, J.; Zhou, X.; Wu, Z.; Wang, D.; Kong, L. Personalized Modeling and Analysis of Mild Adolescent Idiopathic Scoliosis Based on OpenSim. *Yiyong Shengwu Lixue/Journal of Medical Biomechanics* **2023**, *38*, 1146-1153, doi:10.16156/j.1004-7220.2023.06.014.
79. Shetty, A.P.; Raja, D.C. Current trends and advancements in spine surgery. *Journal of Orthopaedics* **2023**, *44*, 31-32, doi:10.1016/j.jor.2023.08.002.
80. Cardozo, M.R.; Singh, S.; Barot, K.; Carey-Ewend, A.; Brehm, S.; Verastegui, G.T.; De La Paz, M.; Hanafy, A.; Olufawo, M.; Yahanda, A.; et al. A360: Augmented reality-assisted percutaneous pedicle screw insertion in the cervical vertebrae: A cadaveric proof-of concept study. *Global Spine Journal* **2023**, *13*, 214S, doi:10.1177/21925682231166108.
81. Sun, W.; Wei, Y.; Zheng, X.; Guo, J.; Yu, L. The application value of 3D finite element analysis technology and 3D printing biological model technology in the precise surgery of degenerative lumbar scoliosis. *Journal of Chinese Physician* **2023**, *25*, 1692-1696, doi:10.3760/cma.j.cn431274-20230330-00421.
82. Tillman, L.C.; Truong, W.H.; Morgan, S.J.; Guillaume, T.J. An in vivo analysis of implanted programmable device interference during magnetically controlled growing rod lengthenings: a story of 129 lengthenings. *Spine Deformity* **2023**, *11*, 1283-1289, doi:10.1007/s43390-023-00709-7.
83. Daroszewski, P.; Huber, J.; Kaczmarek, K.; Janusz, P.; Glówka, P.; Tomaszewski, M.; Domagalska, M.; Kotwicki, T. Comparison of Motor Evoked Potentials Neuromonitoring Following Pre- and Postoperative Transcranial Magnetic Stimulation and Intraoperative Electrical Stimulation in Patients Undergoing Surgical Correction of Idiopathic Scoliosis. *Journal of Clinical Medicine* **2023**, *12*, doi:10.3390/jcm12196312.
84. Garg, D.; Dubey, N.; Goel, P.; Ramoliya, D.; Ganatra, A.; Kotecha, K. Improvisation in Spinal Surgery Using AR (Augmented Reality), MR (Mixed Reality), and VR (Virtual Reality) †. *Engineering Proceedings* **2023**, *59*, doi:10.3390/engproc2023059186.
85. Pahwa, B.; Azad, T.D.; Liu, J.; Ran, K.; Liu, C.J.; Tracz, J.; Sattari, S.A.; Khalifeh, J.M.; Judy, B.F.; Bydon, A.; et al. Assessing the Accuracy of Spinal Instrumentation Using Augmented Reality (AR): A Systematic Review of the Literature and Meta-Analysis. *Journal of Clinical Medicine* **2023**, *12*, doi:10.3390/jcm12216741.
86. Yahara, Y.; Seki, S.; Makino, H.; Futakawa, H.; Kamei, K.; Kawaguchi, Y. Asymmetric Load Transmission Induces Facet Joint Subchondral Sclerosis and Hypertrophy in Patients with Idiopathic Adolescent Scoliosis: Evaluation Using Finite Element Model and Surgical Specimen. *JBMR Plus* **2023**, *7*, doi:10.1002/jbm4.10812.
87. Sakai, D.; Schol, J.; Kawachi, A.; Sako, K.; Hiyama, A.; Katoh, H.; Sato, M.; Watanabe, M. Adolescent Idiopathic Scoliotic Deformity Correction Surgery Assisted by Smart Glasses Can Enhance Correction Outcomes and Accuracy and Also Improve Surgeon Fatigue. *World Neurosurgery* **2023**, *178*, E96-E103, doi:10.1016/j.wneu.2023.06.144.
88. Alvi, M.A.; Kwon, B.K.; Hejrati, N.; Tetreault, L.A.; Evaniew, N.; Skelly, A.C.; Fehlings, M.G. Accuracy of Intraoperative Neuromonitoring in the Diagnosis of Intraoperative

Neurological Decline in the Setting of Spinal Surgery-A Systematic Review and Meta-Analysis. *Global Spine Journal* **2024**, *14*, 105S-149S, doi:10.1177/21925682231196514.
89. Bcharah, G.; Gupta, N.; Panico, N.; Winspear, S.; Bagley, A.; Turnow, M.; D'Amico, R.; Ukachukwu, A.E.K. Innovations in Spine Surgery: A Narrative Review of Current Integrative Technologies. *World Neurosurgery* **2024**, *184*, 127-136, doi:10.1016/j.wneu.2023.12.124.
90. Bhimreddy, M.; Jiang, K.; Weber-Levine, C.; Theodore, N. Computational Modeling, Augmented Reality, and Artificial Intelligence in Spine Surgery. In *Advances in Experimental Medicine and Biology*; Springer: 2024; Volume 1462, pp. 453-464.
91. Daroszewski, P.; Huber, J.; Kaczmarek, K.; Janusz, P.; Główka, P.; Tomaszewski, M.; Kotwicki, T. "Real-Time Neuromonitoring" Increases the Safety and Non-Invasiveness and Shortens the Duration of Idiopathic Scoliosis Surgery. *Journal of Clinical Medicine* **2024**, *13*, doi:10.3390/jcm13051497.
92. De Jesus Encarnacion Ramirez, M.; Chmutin, G.; Nurmukhametov, R.; Soto, G.R.; Kannan, S.; Piavchenko, G.; Nikolenko, V.; Efe, I.E.; Romero, A.R.; Mukengeshay, J.N.; et al. Integrating Augmented Reality in Spine Surgery: Redefining Precision with New Technologies. *Brain Sciences* **2024**, *14*, doi:10.3390/brainsci14070645.
93. Dietz, N.; Alkin, V.; Lieberman, I.; Manista, A.; Kim, T.; Johnson, J.P.; Drazin, D. Scoping review of robotics technology in spinal surgery with highlights of the Annual Seattle Science Foundation Course. *Annals of Translational Medicine* **2024**, *12*, doi:10.21037/atm-24-100.
94. Giraldo, J.P.; Cho, S.S.; Eghrari, N.B.; Dholaria, N.; Farber, S.H.; Ehredt, R.B.; Michaels, C.; Fotias, D.J.; Godzik, J.; Sonntag, V.K.H.; et al. Advances in neurosurgical education: literature review of mixed-reality simulation models and novel mixed-reality spine prototype. *Journal of Neurosurgery-Spine* **2024**, *42*, 385-398, doi:10.3171/2024.8.SPINE24237.
95. Hiranaka, Y.; Takeoka, Y.; Yurube, T.; Tsujimoto, T.; Kanda, Y.; Miyazaki, K.; Ohnishi, H.; Matsuo, T.; Ryu, M.; Kumagai, N.; et al. The Utility and Feasibility of Smart Glasses in Spine Surgery: Minimizing Radiation Exposure During Percutaneous Pedicle Screw Insertion. *Neurospine* **2024**, *21*, 432-439, doi:10.14245/ns.2448090.045.
96. Kanno, H.; Handa, K.; Murotani, M.; Ozawa, H. A Novel Intraoperative CT Navigation System for Spinal Fusion Surgery in Lumbar Degenerative Disease: Accuracy and Safety of Pedicle Screw Placement. *Journal of Clinical Medicine* **2024**, *13*, doi:10.3390/jcm13072105.
97. Nguyen, A.; Dinglasan, M.; Holland, C.; Chung, J.M. THE ACCURACY OF PULSE-TRAIN STIMULATION FOR IMPLANTATION OF PEDICLE SCREWS IN ADOLESCENT SCOLIOSIS. *Journal of Clinical Neurophysiology* **2024**, *41*, e22.
98. Ohashi, M.; Sato, M.; Tashi, H.; Minato, K.; Makino, T.; Kawashima, H. Mixed Reality-Based Navigation for Pedicle Screw Placement: A Preliminary Study Using a 3D-Printed Spine Model. *Cureus Journal of Medical Science* **2024**, *16*, doi:10.7759/cureus.59240.
99. Saghbiny, E.; Leblanc, L.; Harle, A.; Bobbio, C.; Vialle, R.; Morel, G.; Tamadazte, B. Breach Detection in Spine Surgery Based on Cutting Torque. *Ieee Transactions on Medical Robotics and Bionics* **2024**, *6*, 1084-1092, doi:10.1109/TMRB.2024.3421543.

100. Turlip, R.W.; Khela, H.S.; Dagli, M.M.; Chauhan, D.; Ghenbot, Y.; Ahmad, H.S.; Yoon, J.W. Redefining precision: the current and future roles of artificial intelligence in spine surgery. *Artificial Intelligence Surgery* **2024**, *4*, 324-330, doi:10.20517/ais.2024.29.
101. Winkler, D.; Kropla, F.; Busse, M.; Jung, S.; Scholz, S.; Güresir, E.; Gericke, M.; Vychopen, M.; Wach, J.; Grunert, R. Mixed reality for spine surgery: a step into the future with a human cadaveric accuracy study. *Neurosurgical Focus* **2024**, *56*, doi:10.3171/2023.10.FOCUS23619.
102. Leblanc, L.; Saghbini, E.; Da Silva, J.; Harlé, A.; Vafadar, S.; Chandanson, T.; Vialle, R.; Morel, G.; Tamadazte, B. Automatic Spinal Canal Breach Detection During Pedicle Screw Placement. *Ieee Robotics and Automation Letters* **2024**, *9*, 1915-1922, doi:10.1109/LRA.2024.3349947.
103. Tanaka, M.; Schol, J.; Sakai, D.; Sako, K.; Yamamoto, K.; Yanagi, K.; Hiyama, A.; Katoh, H.; Sato, M.; Watanabe, M. Low Radiation Protocol for Intraoperative Robotic C-Arm Can Enhance Adolescent Idiopathic Scoliosis Deformity Correction Accuracy and Safety. *Global Spine Journal* **2024**, *14*, 1504-1514, doi:10.1177/21925682221147867.
104. Lewis, S.J.; Kelly, M.P.; Charalampidis, A.; Swamy, G.; Lenke, L.G. Is a 50% loss in IONM signal the appropriate cut-off in spinal deformity surgery? **2024**, *24*, S85, doi:10.1016/j.spinee.2024.06.066.
105. Misterska, E.; Tomaszewski, M.; Górski, F.; Gapsa, J.; Slysz, A.; Glowacki, M. Assessing the Efficacy of Cognitive-Behavioral Therapy on Body Image in Adolescent Scoliosis Patients Using Virtual Reality. *Journal of Clinical Medicine* **2024**, *13*, doi:10.3390/jcm13216422.
106. Youssef, S.; McDonnell, J.M.; Wilson, K.V.; Turley, L.; Cunniffe, G.; Morris, S.; Darwish, S.; Butler, J.S. Accuracy of augmented reality-assisted pedicle screw placement: a systematic review. *European Spine Journal* **2024**, *33*, 974-984, doi:10.1007/s00586-023-08094-5.
107. Azad, T.D.; Horowitz, M.A.; Tracz, J.A.; Khalifeh, J.M.; Liu, C.J.; Hughes, L.P.; Judy, B.F.; Khan, M.; Bydon, A.; Witham, T.F. Augmented Reality Versus Freehand Spinopelvic Fixation in Spinal Deformity: A Case-Control Study. *Surgical Innovation* **2025**, *32*, 36-45, doi:10.1177/15533506241299887.
108. Dada, A.; Saggi, S.; Ambati, V.S.; Patel, A.; Mummaneni, P.V. Evolution of the Minimally Invasive Surgery Transforaminal Lumbar Interbody Fusion: Where Are We Now? *Neurosurgery* **2025**, *96*, S33-S41, doi:10.1227/neu.0000000000003336.
109. Giraldo, J.P.; Cho, S.S.; Eghrari, N.B.; Dholaria, N.; Farber, S.H.; Ehredt, R.B.; Michaels, C.; Fotias, D.J.; Godzik, J.; Sonntag, V.K.H.; et al. Advances in neurosurgical education: literature review of mixed-reality simulation models and novel mixed-reality spine prototype. *Journal of Neurosurgery: Spine* **2025**, *42*, 385-398, doi:10.3171/2024.8.SPINE24237.
110. Ikwuegbuenyi, C.A.; Inzerillo, S.; Wang, E.; Hussain, I. Strategies for Optimizing Clinical Outcomes in Minimally Invasive Spine Surgery. *Neurosurgery* **2025**, *96*, S139-S147, doi:10.1227/neu.0000000000003331.

111. Kim, J.Y.; Hwang, J.Y.; Park, E.; Nam, H.U.; Cheon, S. Flat-Feet Prediction Based on a Designed Wearable Sensing Shoe and a PCA-Based Deep Neural Network Model. *Ieee Access* **2020**, *8*, 199070-199080, doi:10.1109/ACCESS.2020.3033826.
112. Oppelt, K.; Hogan, A.; Stief, F.; Grützner, P.A.; Trinler, U. Movement Analysis in Orthopedics and Trauma Surgery - Measurement Systems and Clinical Applications. *Zeitschrift Fur Orthopadie Und Unfallchirurgie* **2020**, *158*, 304-317, doi:10.1055/a-0873-1557.
113. Slater, H.; Stinson, J.N.; Jordan, J.E.; Chua, J.; Low, B.; Lalloo, C.; Pham, Q.; Cafazzo, J.A.; Briggs, M. Evaluation of Digital Technologies Tailored to Support Young People's Self-Management of Musculoskeletal Pain: Mixed Methods Study. *Journal of Medical Internet Research* **2020**, *22*, doi:10.2196/18315.
114. Taslimipour, S.; Rojhani-Shirazi, Z.; Hemmati, L.; Rezaei, I. Effects of a Virtual Reality Dance Training Program on Kyphosis Angle and Respiratory Parameters in Young Women With Postural Hyperkyphosis: A Randomized Controlled Clinical Trial. *J Sport Rehabil* **2020**, *30*, 293-299, doi:10.1123/jsr.2019-0303.
115. Zaltieri, M.; Massaroni, C.; Lo Presti, D.; Bravi, M.; Sabbadini, R.; Miccinilli, S.; Sterzi, S.; Formica, D.; Schena, E. A Wearable Device Based on a Fiber Bragg Grating Sensor for Low Back Movements Monitoring. *Sensors* **2020**, *20*, doi:10.3390/s20143825.
116. Ali, A.; Fontanari, V.; Fontana, M.; Schmölz, W. Spinal Deformities and Advancement in Corrective Orthoses. *Bioengineering-Basel* **2021**, *8*, doi:10.3390/bioengineering8010002.
117. Javaid, M.; Haleem, A. Significant advancements of 4D printing in the field of orthopaedics. *Journal of Clinical Orthopaedics and Trauma* **2020**, *11*, S485-S490, doi:10.1016/j.jcot.2020.04.021.
118. Jung, J.Y.; Yang, C.M.; Kim, J.J. Effectiveness of Combined Stretching and Strengthening Exercise Using Rehabilitation Exercise System with a Linear Actuator and MR Damper on Static and Dynamic Sitting Postural Balance: A Feasibility Study. *Applied Sciences-Basel* **2021**, *11*, doi:10.3390/app11167329.
119. Yan, R.G.; Xu, Y.L.; Zhang, H.W.; Jiang, R.A. A ToF-based system for recording human sagittal back shape. *Technology and Health Care* **2021**, *29*, 193-198, doi:10.3233/THC-202397.
120. Willwacher, S.; Koopmann, T.; Dill, S.; Kurz, M.; Bruggemann, G.P. Dorsal muscle fatigue increases thoracic spine curvature in all-out recreational ergometer rowing. *European Journal of Sport Science* **2021**, *21*, 176-182, doi:10.1080/17461391.2020.1737242.
121. Shi, Y.; Zheng, Y.; Zhang, K. Textile-Based Capacitive Pressure Distribution Measurement System for Human Sitting Posture Monitoring. *Journal of Donghua University (English Edition)* **2021**, *38*, 492-497, doi:10.19884/j.1672-5220.202104010.
122. Asadullah, G.M.; Sabyrov, N.; Kamal, M.A.S.; Ali, M.H. Design of a fluid-driven 3D printed spinal posture corrector. *Materials Today-Proceedings* **2021**, *44*, 1555-1559, doi:10.1016/j.matpr.2020.11.774.
123. Odeh, K.; Wu, W.; Taylor, B.; Leasure, J.; Kondrashov, D. In-vitro 3D Analysis of Sacroiliac Joint Kinematics: Primary and Coupled Motions. *Spine* **2021**, *46*, E467-E473, doi:10.1097/BRS.0000000000003841.

124. Adibatti, S.; Sudhindra, K.R.; Joshi Manisha, S. Survey of Advances in Cobb Angle Measurement for Automatic Spine Detection in X-Ray. *WSEAS Transactions on Systems and Control* **2022**, *17 C7 - 49*, 446-457, doi:10.37394/23203.2022.17.49.
125. Willemsen, K.; Magré, J.; Mol, J.; Noordmans, H.J.; Weinans, H.; Hekman, E.E.G.; Kruyt, M.C. Vital Role of In-House 3D Lab to Create Unprecedented Solutions for Challenges in Spinal Surgery, Practical Guidelines and Clinical Case Series. *Journal of Personalized Medicine* **2022**, *12*, doi:10.3390/jpm12030395.
126. Wu, Y.; Lu, Y.; Ma, C.; Zhang, X.; Pan, Z.; Yu, X.; Zhang, Y. Spinal Posture Recognition Device Using Cloud Storage and BP Neural Network Approach Based on Surface Electromyographic Signal. In Proceedings of the Communications in Computer and Information Science, 2022; pp. 507-517.
127. Asadullah, G.M.; Ali, H.; Hashikura, K.; Kamal, M.A.S.; Yamada, K. Development of an Automatic Air-Driven 3D-Printed Spinal Posture Corrector. *Actuators* **2022**, *11*, doi:10.3390/act11070184.
128. Aydın, R.; Alnajjar, F.; Sonoo, M.; Costa Garcia, A.; Takatsune, K.; Shimoda, S. The Influence of Harmonics Filtering for Weak EMG Analysis. In *Biosystems and Biorobotics*; Springer Science and Business Media Deutschland GmbH: 2022; Volume 28, pp. 791-795.
129. Fan, Z.; Hu, X.; Chen, W.M.; Zhang, D.W.; Ma, X. A deep learning based 2-dimensional hip pressure signals analysis method for sitting posture recognition. *Biomedical Signal Processing and Control* **2022**, *73*, doi:10.1016/j.bspc.2021.103432.
130. Hannink, E.; Dawes, H.; Shannon, T.M.L.; Barker, K.L. Validity of sagittal thoracolumbar curvature measurement using a non-radiographic surface topography method. *Spine Deformity* **2022**, *10*, 1299-1306, doi:10.1007/s43390-022-00538-0.
131. Lee, D.; Kim, S.; Park, H.J.; Kim, S.; Shin, D. A Spine Assistive Robot With a Routed Twisted String Actuator and a Flat-Back Alleviation Mechanism for Lumbar-Degenerative Flat Back. *Ieee-Asme Transactions on Mechatronics* **2022**, *27*, 5185-5196, doi:10.1109/TMECH.2022.3175298.
132. Ekambaram, D.; Ponnusamy, V. AI-assisted Physical Therapy for Post-injury Rehabilitation: Current State of the Art. *IEIE Transactions on Smart Processing and Computing* **2023**, *12*, 234-242, doi:10.5573/IEIESPC.2023.12.3.234.
133. Kurland, D.B.; Lau, D.; Kim, N.C.; Ames, C. A bibliometric analysis of patient-reported outcome measures in adult spinal deformity, and the future of patient-centric outcome assessments in the era of predictive analytics. *Seminars in Spine Surgery* **2023**, *35*, doi:10.1016/j.semss.2023.101032.
134. Yan, J.; Wang, A. iGuard: An Intelligent Sitting Posture Monitoring System with Pressure Sensors. In Proceedings of the Proceedings of SPIE - The International Society for Optical Engineering, 2023.
135. Casey, H.L.; Shah, V.V.; Muzyka, D.; McNames, J.; El-Gohary, M.; Sowalsky, K.; Safarpour, D.; Carlson-Kuhta, P.; Schmahmann, J.D.; Rosenthal, L.S.; et al. Standing Balance Conditions and Digital Sway Measures for Clinical Trials of Friedreich's Ataxia. *Movement Disorders* **2024**, *39*, 996-1005, doi:10.1002/mds.29777.

136. Ding, Q.P.; Jiang, J.Y.; Lee, K.M. Reconstruction of a Human Spine Curve in the Sagittal Plane From the Measured Contour of the Human Back. *Ieee Transactions on Instrumentation and Measurement* **2024**, *73*, doi:10.1109/TIM.2024.3378289.
137. Ding, Q.P.; Lee, K.M.; Jiang, J.Y.; Guo, J.J. Subject-Specific Parametric Identification of a Spine-Equivalent Beam Model for Lumbar Force Prediction in Sagittal Plane. *Ieee Transactions on Instrumentation and Measurement* **2024**, *73*, doi:10.1109/TIM.2024.3451588.
138. García-Luna, M.A.; Jimenez-Olmedo, J.M.; Pueo, B.; Manchado, C.; Cortell-Tormo, J.M. Concurrent Validity of the Ergotex Device for Measuring Low Back Posture. *Bioengineering-Basel* **2024**, *11*, doi:10.3390/bioengineering11010098.
139. Gupta, M.C.; Lenke, L.G.; Gupta, S.; Farooqi, A.S.; Boachie-Adjei, O.; Erickson, M.A.; Newton, P.O.; Samdani, A.F.; Shah, S.A.; Shufflebarger, H.L.; et al. Intraoperative neuromonitoring predicts postoperative deficits in severe pediatric spinal deformity patients. *Spine Deformity* **2024**, *12*, 109-118, doi:10.1007/s43390-023-00745-3.
140. Hou, Y.; Liu, H.; Wang, Z. Research status and development trend of smart sitting posture correction garment. *Fangzhi Xuebao/Journal of Textile Research* **2024**, *45*, 250-258, doi:10.13475/j.fzxb.20230606002.
141. Kim, H.; Won, Y.I.; Kang, S.; Choi, Y.; Park, J.H.; Lee, J.; Kim, I.Y.; Chung, C.K. Comparison of Neck Pain and Posture with Spine Angle Tracking System between Static and Dynamic Computer Monitor Use. *Electronics* **2024**, *13*, doi:10.3390/electronics13071363.
142. Kovalenko, A.; Misikov, V.; Kondur, A. Methodology of Botulinum Therapy in the Treatment of Dystonic Scoliosis in Generalized Dystonia (clinical case). *Toxicon* **2024**, *237*, doi:10.1016/j.toxicon.2024.107424.
143. Leoncini, S.; Boasiako, L.; Di Lucia, S.; Beker, A.; Scandurra, V.; Vignoli, A.; Canevini, M.P.; Prato, G.; Nobili, L.; Nicotera, A.G.; et al. 24-h continuous non-invasive multiparameter home monitoring of vitals in patients with Rett syndrome by an innovative wearable technology: evidence of an overlooked chronic fatigue status. *Frontiers in Neurology* **2024**, *15 C7 - 1388506*, doi:10.3389/fneur.2024.1388506.
144. Shaheen, A.; Kazim, H.; Eltawil, M.; Aburukba, R. IoT-Based Solution for Detecting and Monitoring Upper Crossed Syndrome. *Sensors* **2024**, *24*, doi:10.3390/s24010135.
145. Shigematsu, H.; Yasuda, A.; Tangente, R.; Chan, C.Y.W.; Shetty, A.P.; Cheung, J.P.Y.; Hai, Y.; Sakai, D.; Cho, K.J.; Chen, C.W.; et al. Current trends in intraoperative neurophysiological monitoring among Asia-Pacific countries: an Asia-Pacific Spine Society survey. *Asian Spine Journal* **2024**, *18*, 813-821, doi:10.31616/asj.2024.0273.
146. Vos, N.; Kleinendorst, L.; van der Laan, L.; van Uhm, J.; Jansen, P.R.; van Eeghen, A.M.; Maas, S.M.; Mannens, M.M.A.M.; van Haelst, M.M. Evaluation of 100 Dutch cases with 16p11.2 deletion and duplication syndromes; from clinical manifestations towards personalized treatment options. *European Journal of Human Genetics* **2024**, *32*, 1387-1401, doi:10.1038/s41431-024-01601-2.
147. Yang, X.; Fu, R.; Li, P.; Wang, K.; Chen, H. A New Method for Predicting the Porosity of an Interbody Fusion Cage by the Equivalent Material Method. *Journal of Medical and Biological Engineering* **2024**, *44*, 90-98, doi:10.1007/s40846-024-00847-x.

148. Girdler, S.; Cho, B.; Mikhail, C.M.; Cheung, Z.B.; Maza, N.; Cho, S.K.W. Emerging Techniques in Diagnostic Imaging for Idiopathic Scoliosis in Children and Adolescents: A Review of the Literature. *World Neurosurgery* **2020**, *136*, 128-135, doi:10.1016/j.wneu.2020.01.043.
149. Ali, A.; Fontanari, V.; Fontana, M.; Schmölz, W. Spinal Deformities and Advancement in Corrective Orthoses. *Bioengineering (Basel)* **2020**, *8*, doi:10.3390/bioengineering8010002.
150. Huang, R.; Maag, A.; Bhuiyan, M. Augmented reality navigation in spine surgery. In Proceedings of the CITISIA 2020 - IEEE Conference on Innovative Technologies in Intelligent Systems and Industrial Applications, Proceedings C7 - 9371792, 2020.
151. Applebaum, A.; Ference, R.; Cho, W. Evaluating the role of surface topography in the surveillance of scoliosis. *Spine Deformity* **2020**, *8*, 397-404, doi:10.1007/s43390-019-00001-7.
152. Moreira, R.; Teles, A.; Fialho, R.; Baluz, R.; Santos, T.C.; Goulart, R.; Rocha, L.; Silva, F.J.; Gupta, N.; Bastos, V.H.; et al. Mobile Applications for Assessing Human Posture: A Systematic Literature Review. *Electronics* **2020**, *9*, doi:10.3390/electronics9081196.
153. Oppelt, K.; Hogan, A.; Stief, F.; Grützner, P.A.; Trinler, U. Movement Analysis in Orthopedics and Trauma Surgery - Measurement Systems and Clinical Applications. *Zeitschrift fur Orthopadie und Unfallchirurgie* **2020**, *158*, 304-316, doi:10.1055/a-0873-1557.
154. Yan, D.; Vassar, R. Neuromuscular electrical stimulation for motor recovery in pediatric neurological conditions: a scoping review. *Developmental Medicine and Child Neurology* **2021**, *63*, 1394-1401, doi:10.1111/dmcn.14974.
155. Kamalapathy, P.N.; Karhade, A.V.; Tobert, D.; Schwab, J.H. Artificial Intelligence in Adult Spinal Deformity. In *Acta Neurochirurgica, Supplementum*; Springer Science and Business Media Deutschland GmbH: 2022; Volume 134, pp. 313-318.
156. Li, X.; Huo, Z.; Hu, Z.; Lam, T.P.; Cheng, J.C.Y.; Chung, V.C.; Yip, B.H.K. Which interventions may improve bracing compliance in adolescent idiopathic scoliosis? A systematic review and meta-analysis. *PLoS One* **2022**, *17*, e0271612, doi:10.1371/journal.pone.0271612.
157. Ameta, K.L.; Solanki, V.S.; Singh, V.; Devi, A.P.; Chundawat, R.S.; Haque, S. Critical appraisal and systematic review of 3D & 4D printing in sustainable and environment-friendly smart manufacturing technologies. *Sustainable Materials and Technologies* **2022**, *34*, doi:10.1016/j.susmat.2022.e00481.
158. Syamlan, A.; Fathurachman; Denis, K.; Vander Poorten, E.; Pramujati, B.; Tjahjowidodo, T. Haptic/virtual reality orthopedic surgical simulators: a literature review. *Virtual Reality* **2022**, *26*, 1795-1825, doi:10.1007/s10055-022-00666-y.
159. Karandikar, P.; Massaad, E.; Hadzipasic, M.; Kiapour, A.; Joshi, R.S.; Shankar, G.M.; Shin, J.H. Machine Learning Applications of Surgical Imaging for the Diagnosis and Treatment of Spine Disorders: Current State of the Art. *Neurosurgery* **2022**, *90*, 372-382, doi:10.1227/NEU.0000000000001853.
160. Ng, P.T.T.; Claus, A.; Izatt, M.T.; Pivonka, P.; Tucker, K. Is spinal neuromuscular function asymmetrical in adolescents with idiopathic scoliosis compared to those without scoliosis?: A narrative review of surface EMG studies. *Journal of Electromyography and Kinesiology* **2022**, *63*, doi:10.1016/j.jelekin.2022.102640.

161. Grivas, T.B.; Negrini, S.; Aubin, C.E.; Aulisa, A.G.; De Mauroy, J.C.; Donzelli, S.; Hresko, M.T.; Kotwicki, T.; Lou, E.; Maruyama, T.; et al. Nonoperative management of adolescent idiopathic scoliosis (AIS) using braces. *Prosthetics and Orthotics International* **2022**, *46*, 383-391, doi:10.1097/PXR.0000000000000117.
162. Webster, T. 25 years of commercializing nanomedicine: from tissue engineering to fighting COVID. *Tissue Engineering - Part A* **2022**, *28*, 335, doi:10.1089/ten.tea.2022.29037.abstract.index.
163. Bottino, L.; Settino, M.; Cannataro, M. Distributed ICT solutions for scoliosis management. In Proceedings of the Proceedings - 2023 31st Euromicro International Conference on Parallel, Distributed and Network-Based Processing, PDP 2023, 2023; pp. 258-262.
164. Cerillo, J.L.; Becsey, A.N.; Sanghadia, C.P.; Root, K.T.; Lucke-Wold, B. Spine Bracing: When to Utilize-A Narrative Review. *Biomechanics* **2023**, *3*, 136-154, doi:10.3390/biomechanics3010013.
165. Cordani, C.; Malisano, L.; Febbo, F.; Giranio, G.; Del Furia, M.J.; Donzelli, S.; Negrini, S. Influence of Specific Interventions on Bracing Compliance in Adolescents with Idiopathic Scoliosis-A Systematic Review of Papers Including Sensors' Monitoring. *Sensors* **2023**, *23*, doi:10.3390/s23177660.
166. Kurland, D.; Bi, C.; Ber, R.; Kondziolka, D.; Lau, D.; Pacione, D.; Frempong-Boadu, A.; Laufer, I.; Oermann, E. P464: Smartphones, wearables, and digital biomarkers: The evolution of spine care outcomes research. *Global Spine Journal* **2023**, *13*, 458S, doi:10.1177/21925682231166109.
167. Khan, M.A.; Pogonchenkova, I.V.; Talkovsky, E.M.; Vybornov, D.Y.; Kuyantseva, L.V.; Tarasov, N.I.; Koroteev, V.V. Electrical Stimulation Methods for Scoliosis in Children: a Literature Review. *Vestnik Vosstanovitel'noj Mediciny* **2024**, *23*, 116-124, doi:10.38025/2078-1962-2024-23-5-116-124.
168. Paramento, M.; Passarotto, E.; Maccarone, M.C.; Agostini, M.; Contessa, P.; Rubega, M.; Formaggio, E.; Masiero, S. Neurophysiological, balance and motion evidence in adolescent idiopathic scoliosis: A systematic review. *Plos One* **2024**, *19*, doi:10.1371/journal.pone.0303086.
169. Kim, H.; Chang, B.S.; Chang, S.Y. Current issues in the treatment of adolescent idiopathic scoliosis: a comprehensive narrative review. *Asian Spine Journal* **2024**, *18*, 731-742, doi:10.31616/asj.2024.0367.
170. Cheng, F.; Lu, L.; Sun, M.; Wang, X.; Wang, Y. Non-invasive Scoliosis Assessment in Adolescents. In Proceedings of the Lecture Notes of the Institute for Computer Sciences, Social-Informatics and Telecommunications Engineering, LNICST, 2024; pp. 221-230.
171. Agravante, A.N.L.; Carpeso, I.R.T.; Guillermo, K.M.S.; Lim, K.D.C.; Lim, R.A.U.; Lopez, N.N.C.; Baldovino, R.G. Emerging Technologies of Sensor-Based Assistive Devices for Spinal Position Monitoring: A Review. In Proceedings of the IBIOMED 2024 - Proceedings of the 5th International Conference on Biomedical Engineering 2024, 2024; pp. 23-28.
172. Alvi, M.A.; Kwon, B.K.; Hejrati, N.; Tetreault, L.A.; Evaniew, N.; Skelly, A.C.; Fehlings, M.G. Accuracy of Intraoperative Neuromonitoring in the Diagnosis of Intraoperative

Neurological Decline in the Setting of Spinal Surgery—A Systematic Review and Meta-Analysis. *Global Spine Journal* **2024**, *14*, 105S-149S, doi:10.1177/21925682231196514.
173. Lee, S.; Jung, J.Y.; Mahatthanatrakul, A.; Kim, J.S. Artificial Intelligence in Spinal Imaging and Patient Care: A Review of Recent Advances. *Neurospine* **2024**, *21*, 474-486, doi:10.14245/ns.2448388.194.
174. Karimi, M.T.; Kavyani, M. Evaluation of the effectiveness of soft braces on idiopathic scoliosis: A review of literature. *Journal of Orthopaedics Trauma and Rehabilitation* **2024**, *31*, 85-92, doi:10.1177/22104917231191800.
175. Förstl, N.; Adler, I.; Süss, F.; Dendorfer, S. Technologies for Evaluation of Pelvic Floor Functionality: A Systematic Review. *Sensors* **2024**, *24*, doi:10.3390/s24124001.
176. Ratnaparkhi, A.; Beckett, J. Digital Phenotyping, Wearables, and Outcomes. *Neurosurgery Clinics of North America* **2024**, *35*, 235-241, doi:10.1016/j.nec.2023.11.009.
177. Luo, C.; Wu, H.; Liu, W.; Luo, Y.; Jie, Y.; Ma, C.Z.H.; Wong, M. The Biomechanics of Spinal Orthoses for Adolescent Idiopathic Scoliosis: A Systematic Review of the Controlling Forces. *Bioengineering* **2024**, *11*, doi:10.3390/bioengineering11121242.
178. Kaelin, A.J. Adolescent idiopathic scoliosis: indications for bracing and conservative treatments. *Annals of Translational Medicine* **2020**, *8*, doi:10.21037/atm.2019.09.69.
179. Tapp, A.; Payer, C.; Schmid, J.; Polanco, M.; Kumi, I.; Bawab, S.; Ringleb, S.; St. Remy, C.; Bennett, J.; Kakar, R.S.; et al. Generation of Patient-Specific, Ligamentoskeletal, Finite Element Meshes for Scoliosis Correction Planning. In Proceedings of the Lecture Notes in Computer Science (including subseries Lecture Notes in Artificial Intelligence and Lecture Notes in Bioinformatics), 2021; pp. 13-23.
180. Negrini, S.; Aulisa, A.G.; Cerny, P.; de Mauroy, J.C.; McAviney, J.; Mills, A.; Donzelli, S.; Grivas, T.B.; Hresko, M.T.; Kotwicki, T.; et al. The classification of scoliosis braces developed by SOSORT with SRS, ISPO, and POSNA and approved by ESPRM. *European Spine Journal* **2022**, *31*, 980-989, doi:10.1007/s00586-022-07131-z.
181. Te Hennepe, N.; Steegh, V.; Pouw, M.H.; Roukema, J.; De Kleuver, M.; Van Hooff, M.L. Pulmonary function in patients with adolescent idiopathic scoliosis: an explorative study of a wearable smart shirt as a measurement instrument. *Spine Deformity* **2025**, *13*, 101-110, doi:10.1007/s43390-024-00938-4.
